# Comparative In Vitro Deposition Analysis of Formoterol, Glycopyrronium, and Tiotropium Delivered via Capsule-Based DPI

**DOI:** 10.3390/pharmaceutics17091089

**Published:** 2025-08-22

**Authors:** Adam Sikora, Joanna Chałupka, Kinga Lewandowska, Paulina Drapińska, Michał Piotr Marszałł

**Affiliations:** 1 Department of Medicinal Chemistry, Faculty of Pharmacy, Collegium Medicum in Bydgoszcz, Nicolaus Copernicus University in Toruń, Dr. A. Jurasza 2, 85-089 Bydgoszcz, Poland; joanna.chalupka@cm.umk.pl (J.C.); kinga.lewandowska@cm.umk.pl (K.L.); mmars@cm.umk.pl (M.P.M.); 2 Medical Biotechnology and Laboratory Medicine, Department of Pharmaceutical Technology, Faculty of Pharmacy, Pomeranian Medical University in Szczecin, Pl. Polskiego Czerwonego Krzyża 1, 71-244 Szczecin, Poland; paulina.drapinska@pum.edu.pl

**Keywords:** dry powder inhalers (DPIs), fine particle dose (FPD), aerodynamic particle size distribution (APSD), pulmonary drug delivery

## Abstract

Dry powder inhalers (DPIs) are the mainstay in the treatment of obstructive pulmonary diseases. However, the performance of DPI formulations is highly dependent on the used inhaler device and the patient’s inspiratory effort. This study aimed to evaluate and compare the aerosolization behavior of three commercially available capsule-based DPI medications—formoterol (Foradil^®^), glycopyrronium (Seebri^®^ Breezhaler), and tiotropium (Spiriva^®^)—delivered using three different capsule-based inhalers (Aerolizer, Breezhaler, and Handihaler), under varying flow conditions. **Methods:** The aerodynamic performance of each formulation–inhaler combination was assessed using the Next-Generation Impactor (NGI) and Dosage Unit Sampling Apparatus (DUSA) methodology. Fine particle dose (FPD) and aerodynamic particle size distribution (APSD) were determined at fixed flow rates of 15, 30, 60, and 100 L/min, as well as at inhaler-specific flow rates corresponding to a 4 kPa pressure drop. Chromatographic quantification of active ingredients was performed using validated HPLC methods specific to each drug. **Results:** The FPD values increased consistently with higher flow rates across all tested formulations and inhalers. At a 4 kPa pressure drop, Aerolizer and Breezhaler achieved significantly higher FPDs compared to Handihaler. Notably, in some instances, non-dedicated inhalers produced greater respirable fractions than the originally intended devices. APSD profiles revealed that drug deposition shifted toward smaller NGI stages at higher inspiratory flows, supporting enhanced deep lung delivery potential under optimal conditions. **Conclusions:** Device resistance, capsule orientation, and piercing mechanics substantially influence drug aerosolization. Although non-dedicated inhalers may offer improved FPDs in vitro, clinical use should adhere to approved drug–device combinations, as these have been validated for efficacy and safety under real-world conditions.

## 1. Introduction

Asthma and chronic obstructive pulmonary disease (COPD) are significant public health challenges, affecting over 500 million individuals worldwide and ranking among the leading causes of morbidity and mortality [1,2]. These conditions impose substantial healthcare and economic burdens, particularly due to recurrent exacerbations, hospitalizations, and reduced productivity. Inhaled medications, which deliver drugs directly to the respiratory tract, remain the cornerstone of treatment. This localized approach minimizes systemic side effects while maximizing therapeutic efficacy, making inhalation therapy the standard in managing asthma and COPD.

Among the commonly prescribed inhaled drugs are inhaled corticosteroids (e.g., budesonide, fluticasone), long-acting beta-agonists (e.g., formoterol), and long-acting muscarinic antagonists (e.g., tiotropium, glycopyrronium). These medications are delivered via various types of dry powder inhalers (DPIs), including the Cyclohaler, Aerolizer, and Handihaler, which have been developed to ensure efficient drug dispersion and deposition in the lungs. Each device employs distinct mechanisms for aerosol generation, ranging from capsule puncturing systems to rotary dispersion chambers. However, the success of these devices heavily depends on the user’s ability to perform correct inhalation maneuvers, a challenge that significantly impacts clinical outcomes [3,4,5].

Despite the technological advancements in inhaler design, incorrect inhaler use remains a pervasive issue among patients with asthma and COPD. Errors such as inadequate inhalation flow, improper loading of capsules, and the use of non-dedicated inhalers for specific medications are frequently observed [6,7,8,9,10,11]. Such errors compromise the aerodynamic particle size distribution (APSD) of the delivered drug, leading to suboptimal lung deposition and reduced therapeutic efficacy. Moreover, the misuse of inhalers can result in dose variability and drug wastage, further complicating disease management [12,13,14,15,16].

The Breezhaler, Aerolizer, and Handihaler are among the most commonly used DPIs for administering a wide range of inhaled drugs. These devices differ in their internal structures, capsule puncturing mechanisms, and airflow characteristics, all of which influence the drug’s dispersion and APSD. Understanding the impact of these differences on the delivered dose and particle size distribution is critical for optimizing inhalation therapy and educating patients on proper inhaler use.

This study aimed to assess the impact of three commercially available DPIs—Breezhaler, Aerolizer, and Handihaler—on the APSD and dose uniformity of inhaled medications containing formoterol, tiotropium, and glycopyrronium. The aerodynamic performance of these medications was evaluated using the Next-Generation Impactor (NGI), a widely recognized tool for simulating drug deposition across the respiratory tract. The NGI enables precise separation and quantification of drug particles based on their aerodynamic diameter, providing valuable insights into lung deposition patterns [17].

Quantitative analyses of the collected samples were conducted using Ultra-Performance Liquid Chromatography (UPLC), a state-of-the-art analytical technique that ensures high sensitivity, precision, and accuracy. Each UPLC method was meticulously optimized and validated according to International Council for Harmonisation (ICH) guidelines, ensuring robust and reliable data. The combination of NGI, DUSA, and UPLC methodologies enabled a comprehensive evaluation of the inhalers’ performance, highlighting the potential clinical consequences of using non-dedicated devices.

The findings of this study have significant implications for the management of asthma and COPD. By demonstrating the influence of inhaler selection on drug delivery performance, this research underscores the critical importance of patient education and device optimization. Proper inhaler use is essential for achieving optimal therapeutic outcomes, reducing exacerbation rates, and minimizing healthcare costs. Moreover, this study highlights the need for healthcare providers to emphasize the correct matching of medications with their respective inhalation devices to ensure consistent and effective treatment [18,19].

By addressing these challenges, this work aims to contribute to the growing body of evidence supporting personalized inhalation therapy, ultimately improving patient adherence and disease control. Future research should focus on developing intuitive inhaler designs and implementing comprehensive training programs to mitigate the high prevalence of inhaler misuse [20].

## 2. Materials and Methods

### 2.1. Chemicals

Formic acid and ammonium formate were purchased from Sigma-Aldrich (St. Louis, MO, USA). Methanol (HPLC grade, 99.9%) was procured from Honeywell (Morris Plains, NJ, USA). Ultrapurified water was generated using a Milli-Q Water Purification System (Merck Millipore, Burlington, MA, USA).

Foradil^®^ (12 µg formoterol fumarate per dose; equivalent to 12.5 µg formoterol fumarate dihydrate) is a capsule-based dry powder inhalation product manufactured by Novartis (Basel, Switzerland). Each hard gelatin capsule contains micronized formoterol fumarate blended with lactose monohydrate as a carrier. The formulation does not contain additional excipients such as lubricants or dispersing agents.

Seebri^®^ Breezhaler (44 µg glycopyrronium bromide per dose; equivalent to 55 µg glycopyrronium) is manufactured by Novartis (Basel, Switzerland). Each capsule contains micronized glycopyrronium bromide blended with inhalation-grade lactose monohydrate and a small amount of magnesium stearate, which serves as a dispersibility enhancer and may influence de-agglomeration behavior during inhalation.

Spiriva^®^ (18 µg tiotropium bromide monohydrate per dose; equivalent to 22.5 µg tiotropium bromide) is a capsule-based inhalation powder manufactured by Boehringer Ingelheim (Ingelheim am Rhein, Germany). Each capsule contains micronized tiotropium bromide monohydrate blended with lactose monohydrate as the carrier. No additional excipients are listed in the product’s summary of product characteristics (SmPC).

All products were obtained from commercial sources and used as received. Their qualitative and quantitative compositions were confirmed based on publicly available regulatory documentation, including the European Medicines Agency (EMA) product information and summary of product characteristics (SmPC). These compositional details are critical for interpreting differences in aerosol performance, as the presence of lubricants such as magnesium stearate and the physicochemical properties of the lactose carrier can significantly influence powder dispersion and aerodynamic behavior during inhalation.

### 2.2. Instrumentation

Sample preparation and analysis were conducted using a Shimadzu HPLC system (Kyoto, Japan) composed of dual solvent feed pumps with a gradient system (LC-30AD), degasser (DGU-20A5R), autosampler (SIL-30AC), column oven (CTO-20AC), UV-VIS detector (SPD-20AD), and Kinetex C18 column (150 × 4.6 mm; Phenomenex, Torrance, CA, USA).

A Milli-Q Water Purification System (Merck Millipore, Burlington, MA, USA) was used for ultrapure water preparation. An XA 82/220.R2 analytical balance (Radwag, Radom, Poland) ensured precise measurements. The Unimax 1010 shaker (Heidolph, Schwabach, Germany) was used for controlled incubations.

Aerodynamic particle size distribution (APSD) analyses were performed using a Next-Generation Impactor (NGI) (Copley Scientific, Nottingham, UK), connected to an HCP5 High-Capacity Vacuum Pump (Copley Scientific, UK) equipped with a Critical Flow Controller Model TPK-R™ (Copley Scientific, UK). This configuration ensured stable and reproducible airflow rates, in accordance with compendial testing procedures for inhalation products (Ph. Eur. 11.0; USP <601>). A digital flow meter and standardized mouthpiece adapters (also from Copley Scientific) were used for flow calibration and device connection. All pipetting procedures were performed using Tacta adjustable-volume pipettes (Sartorius Biohit, Göttingen, Germany) and the Eppendorf Multipette E3 system (Eppendorf, Hamburg, Germany). 

Each piece of glass we used was dried in an oven overnight before being cooled with a stream of nitrogen. 

### 2.3. APSD Analysis

All aerodynamic assessments were performed under steady-state flow conditions, as defined by the European Pharmacopoeia (Ph. Eur. 11.0) and the United States Pharmacopeia (USP <601>). The airflow was precisely regulated using a calibrated critical flow controller and vacuum pump system, enabling predefined constant flow rates (15, 30, 60, and 100 L/min) or standardized pressure drops (4 kPa), depending on the experimental setup. While these parameters do not replicate dynamic patient-specific inspiratory profiles—such as ramp-up flow characteristics—they provide a reproducible and pharmacopeia-compliant basis for comparative in vitro assessment of dry powder inhalers. This standardized approach ensures consistency and comparability of fine particle dose (FPD) and aerodynamic particle size distribution (APSD) across devices.

Three commercially available capsule-based dry powder inhalers (DPIs)—Breezhaler^®^, HandiHaler^®^, and Aerolizer^®^—were selected for in vitro APSD assessments of three different formulations: Spiriva^®^, Seebri^®^, and Foradil^®^. Prior to analysis, each capsule was individually weighed on an analytical balance to determine its initial mass.

The experimental setup consisted of a vacuum pump connected to a NGI, with the flow rate precisely set at various airflow velocities in the range of 15–120 L/min and the inhalation time adjusted so that the total air volume remained 4 L. For instance, at 100 L/min, the air was drawn for 2.4 s to simulate a typical forced inhalation with 4 L of inhaled volume.

Each capsule was inserted into the appropriate DPI, which was held upright, and actuated by simultaneously pressing both buttons to pierce the capsule using the integrated needle system. The inhaler was then mounted into a standardized mouthpiece adapter connected to the NGI inlet.

Upon activation of the vacuum pump, air was drawn through the inhaler at the predefined flow rate, aerosolizing the powder from the capsule. The emitted particles were subsequently deposited on specific NGI stages according to their aerodynamic diameter. After actuation, the capsule was removed and reweighed to calculate the emitted dose (ED) based on the mass difference before and after inhalation.

This procedure was repeated for ten capsules per drug–device combination to ensure adequate repeatability. Following aerosol deposition, each NGI stage and the mouthpiece adapter were rinsed with 5 mL of methanol to extract the deposited drug substance. The NGI collection tray was placed on a laboratory shaker and agitated for 5 min at 50 rpm. Similarly, the inlet port was sealed with rubber stoppers and shaken using a dedicated Copley induction port shaker under the same conditions.

Approximately 1 mL of extract was withdrawn from each compartment using a syringe, filtered through 0.45 µm hydrophobic membrane filters, and transferred to HPLC vials. These samples were analyzed to quantify the deposition of the drug on each impactor stage.

To assess the influence of airflow rate on drug aerosolization, the experimental procedure was repeated at 60 L/min (inhalation time: 4 s), 30 L/min (8 s), and 15 L/min (16 s), as well as at a standardized pressure drop of 4 kPa (flow rate depending on the device resistance). Each drug formulation was tested across all selected DPIs to allow comprehensive comparison of aerodynamic performance, including off-label device combinations.

Quantitative analysis of drug deposition on each NGI stage was performed as described in the analytical section. The fine particle dose (FPD)—defined as the dose fraction with an aerodynamic diameter < 5 µm—was calculated for each setup to estimate the respirable fraction likely to reach the lower respiratory tract.

### 2.4. Chromatographic Analysis

Quantitative analyses of formoterol fumarate, tiotropium bromide, and glycopyrronium bromide deposited on individual stages of the NGI and in the induction port were performed using high-performance liquid chromatography (HPLC). All analyses were carried out using a Shimadzu liquid chromatograph equipped with a UV/VIS detector, autosampler, and column thermostat. The detailed chromatographic conditions are placed in Table 1. 

For the quantification of formoterol, an isocratic elution method was employed. The mobile phase consisted of two components: phase A—methanol with 0.1% formic acid (42.5%, *v*/*v*); phase B—water containing 10 mM ammonium formate and 0.1% formic acid (57.5%, *v*/*v*). The separation was performed on a C18 reversed-phase column maintained at 30 °C. The flow rate of the mobile phase was set at 1.0 mL/min. The injection volume was 30 µL, and the analysis time per sample was 7.5 min. Detection was carried out at a wavelength of 230 nm.

For the analysis of tiotropium, the mobile phase was composed of 60% aqueous 10 mM ammonium formate with 0.1% formic acid and 40% methanol with 0.1% formic acid. The chromatographic separation was performed on a C18 column at 20 °C, with a mobile phase flow rate of 1.0 mL/min. A 30 µL volume of each sample was injected, and detection was conducted at 230 nm. The retention time of tiotropium was approximately 1.58 min. Prior to sample injection, the system was equilibrated for at least 20 min under the specified conditions. After analysis, the system was flushed with methanol followed by water to prepare for subsequent runs or shutdown.

For the quantification of glycopyrronium, the mobile phase consisted of 45% methanol with 0.1% formic acid and 55% water containing 10 mM ammonium formate (prepared by dissolving 0.63 g ammonium formate in 1 L of water) with 0.1% formic acid. Chromatographic separation was performed using a Kinetex C18 column (150 mm × 4.6 mm, 5 µm particle size) at a flow rate of 1.0 mL/min. The injection volume was 30 µL and the analysis time was 5 min. Detection was carried out at 230 nm.

In all cases, sample vials were loaded into the autosampler, and data were acquired and processed using integrated chromatographic software. The resulting chromatograms were used to quantify drug deposition at each NGI stage and calculate respirable dose metrics, including the fine particle dose (FPD).

All chromatographic methods were internally validated according to ICH Q2(R1) guidelines. Parameters such as linearity (R^2^ > 0.999), accuracy (recoveries within 98–102%), precision (RSD < 2%), LOD, and LOQ were established and met the acceptance criteria.

### 2.5. Statistical Analysis

The statistical analysis was conducted to determine whether the observed differences in fine particle dose of tested formulations between the three dry powder inhalers (DPIs)—Aerolizer, Breezhaler^®^, and Handihaler^®^—were statistically significant under different flow conditions. For each of the three tested inhalation products (formoterol, glycopyrronium, and tiotropium), the influence of inhaler type on FPD was assessed at five inspiratory flow rates: 15 L/min, 30 L/min, 60 L/min, 100 L/min, and the flow corresponding to a pressure drop of approximately 4 kPa (i.e., ~110 L/min for Aerolizer, ~115 L/min for Breezhaler^®^, and ~55 L/min for Handihaler^®^).

Statistical analyses were performed using the Statistica 13.3 software package (StatSoft Inc., Kraków, Poland). For each drug and each flow rate, a one-way analysis of variance (ANOVA) was performed, with the type of inhaler as the independent variable and the FPD as the dependent variable. Prior to ANOVA, assumptions of normal distribution and homogeneity of variances were verified using the Shapiro–Wilk and Levene’s tests, respectively.

Where the ANOVA indicated statistically significant differences (*p* < 0.05), a Tukey’s Honest Significant Difference (HSD) post hoc test was applied to identify specific pairwise differences between inhalers. This post hoc procedure enabled determination of whether any of the devices produced significantly higher or lower FPD values compared to the others at a given flow rate. All statistical tests were two-sided, and significance was accepted at *p* < 0.05.

The results of the ANOVA and Tukey’s HSD tests are provided in the Supplementary Material (Figures S1–S15 and Tables S1–S15). Each comparison was performed separately for each drug substance and inspiratory flow condition. Outcomes of the analysis are discussed in the Results section in the context of aerodynamic performance.

## 3. Results

Three commercially available capsule-based inhalation products—formoterol fumarate (Foradil^®^), glycopyrronium bromide (Seebri^®^ Breezhaler), and tiotropium bromide (Spiriva^®^)—were evaluated using the DUSA and the NGI across a range of controlled airflow rates: 15, 30, 60, and 100 L/min, as well as at individual airflow rates corresponding to a pressure drop of 4 kPa for each inhaler. For all formulations, delivered dose (DD) (Table 2), fine particle dose (FPD), and aerodynamic particle size distribution (APSD) were determined. Based on the obtained data, FPD versus airflow rate curves were generated to illustrate the dependence of aerodynamic performance on inspiratory effort. These analyses allowed for the comparative assessment of how each inhaler–formulation combination delivers the active pharmaceutical ingredient under varying flow conditions and to what extent they support optimal pulmonary deposition.

All tested active pharmaceutical ingredients (formoterol fumarate, glycopyrronium bromide, and tiotropium bromide monohydrate) were delivered using capsule-based dry powder inhalers (DPIs): Aerolizer^®^, Breezhaler^®^, and Handihaler^®^. These devices differ significantly in their structural design, airflow resistance, and capsule orientation, which collectively influence aerosol generation and drug deposition profiles. Aerolizer^®^ and Breezhaler^®^ are classified as low-resistance inhalers, enabling efficient powder dispersion even at relatively modest inspiratory flow rates. In contrast, Handihaler^®^ is characterized by medium-to-high airflow resistance and requires greater patient effort to achieve effective powder de-aggregation. Another critical distinction lies in capsule orientation: Aerolizer^®^ and Breezhaler^®^ hold the capsule in a horizontal position, whereas Handihaler^®^ orients it vertically. Additionally, Aerolizer^®^ features a system of eight piercing needles designed to enhance capsule perforation and rotation during inhalation, while both Breezhaler^®^ and Handihaler^®^ employ two piercing needles, resulting in distinct internal airflow patterns due to their differing geometries and resistance profiles. These structural differences are directly reflected in the aerodynamic performance and clinical usability of each drug–device combination.

To characterize these effects, the aerosolization performance of each formulation–device pairing was assessed using a Next-Generation Impactor (NGI) at four standardized volumetric flow rates (15, 30, 60, and 100 L/min), as well as a fifth, device-specific flow corresponding to a pressure drop of 4 kPa: approximately 110 L/min for Aerolizer^®^, 115 L/min for Breezhaler^®^, and 55 L/min for Handihaler^®^. For each condition, stage-specific cut-off diameters (d_50_) were calculated to enable accurate classification of aerodynamic particle size. The fine particle dose (FPD), defined as the mass of particles with aerodynamic diameter <5 µm, was then determined by summing the deposited mass on the corresponding NGI stages. This approach allowed for robust comparison of de-aggregation efficiency and respirable dose delivery across devices and flow regimes for each tested drug product.

### 3.1. Influence of Flow Rate on the Fine Particle Dose (FPD) of Formoterol

**Figure 1:**
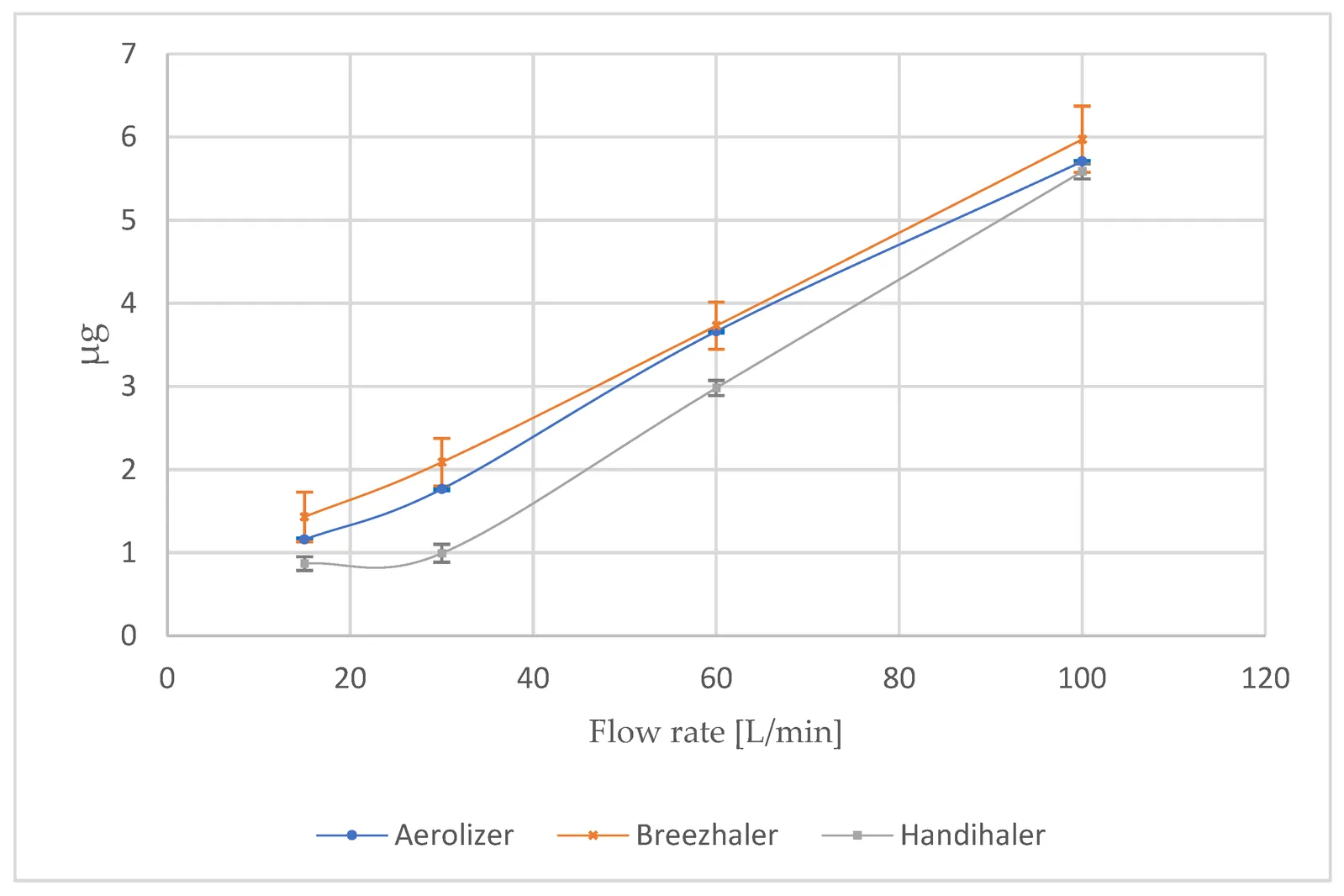
Fine particle dose (<5 µm) of formoterol emitted from Foradil^®^ capsules using Aerolizer^®^, Breezhaler^®^, and Handihaler^®^ at different flow rates.

At intermediate and higher flows, the differences between devices narrowed, which is presented in the Figure 1. At 60 L/min, all inhalers demonstrated a marked increase in FPD: 3.66 ± 0.02 µg (Aerolizer), 3.73 ± 0.28 µg (Breezhaler), and 2.98 ± 0.09 µg (Handihaler). At 100 L/min, the values converged further: 5.71 ± 0.01 µg (Aerolizer), 5.97 ± 0.40 µg (Breezhaler), and 5.59 ± 0.09 µg (Handihaler).

The most revealing condition was the device-specific 4 kPa pressure drop, which represents the clinically relevant inspiratory effort for each device (Table 3). Under these conditions, the low-resistance devices maintained optimal respirable output: 6.15 ± 0.04 µg for Aerolizer (110 L/min) and 6.49 ± 0.01 µg for Breezhaler (115 L/min). In contrast, Handihaler’s FPD was substantially lower (2.08 ± 0.01 µg) at a flow of only ~55 L/min, despite the same nominal dose and analytical setup. This drop reflects the device’s higher intrinsic resistance and limited de-agglomeration efficiency at the reduced flow required to generate the same pressure drop.

### 3.2. Aerodynamic Particle Size Distribution (APSD) of Formoterol

**Figure 2:**
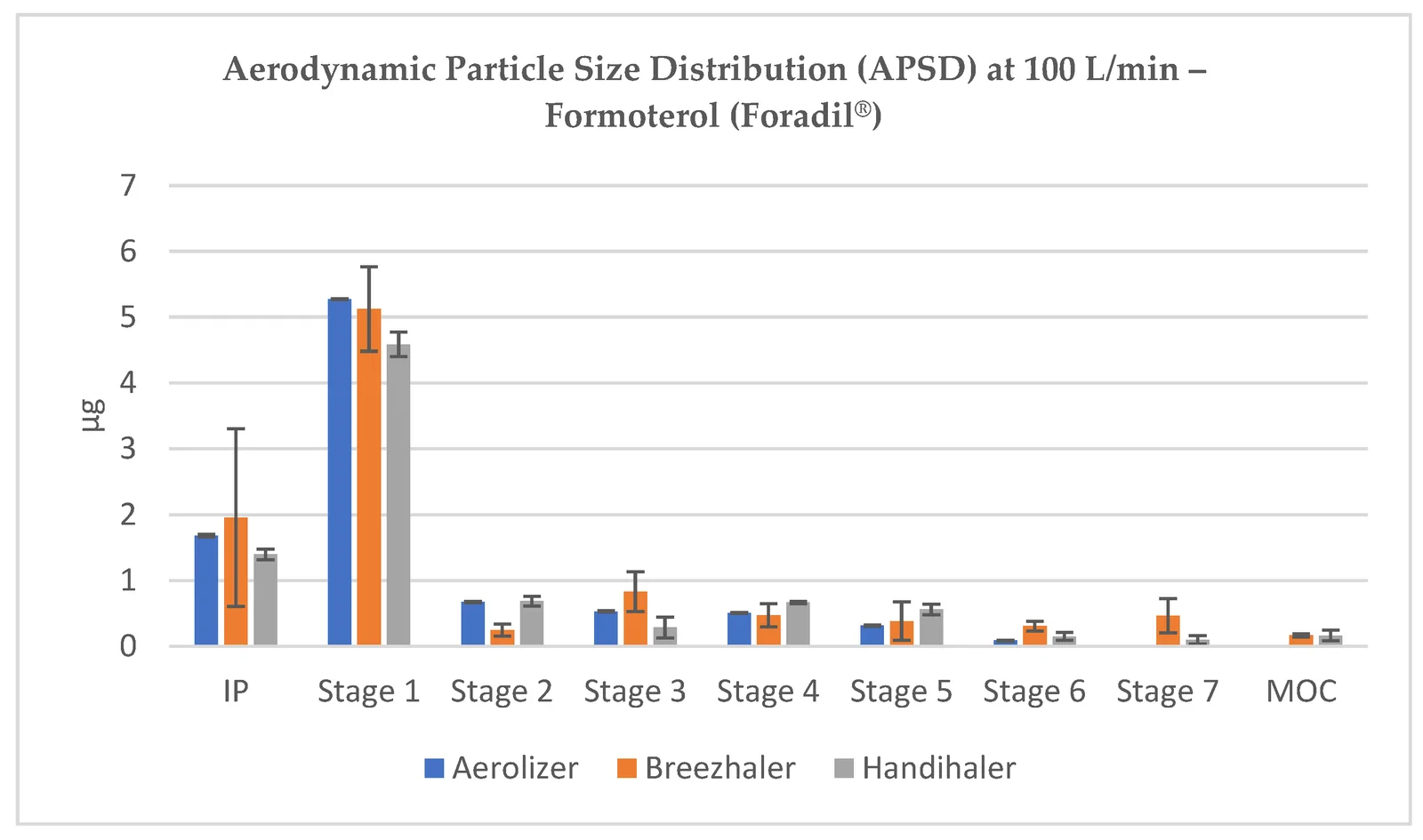
Aerodynamic particle size distribution (APSD) of formoterol from Foradil^®^ capsules at 100 L/min using Aerolizer^®^, Breezhaler^®^, and Handihaler^®^. Mean ± SD (*n* = 10). One-way ANOVA + Tukey’s HSD for fine particle dose: all comparisons ns.

The NGI stage-by-stage deposition profiles revealed consistent trends across flow conditions. At 100 L/min, all inhalers achieved substantial drug deposition across the lower NGI stages, with visible peaks in Stage 3 and Stage 4, confirming the formation of particles within the optimal aerodynamic diameter range (~1–3 µm). Stage 1 remained dominant in terms of mass, reflecting residual coarse material or agglomerates, but the respirable fraction accounted for a significant portion of the emitted dose (Figure 2).

**Figure 3:**
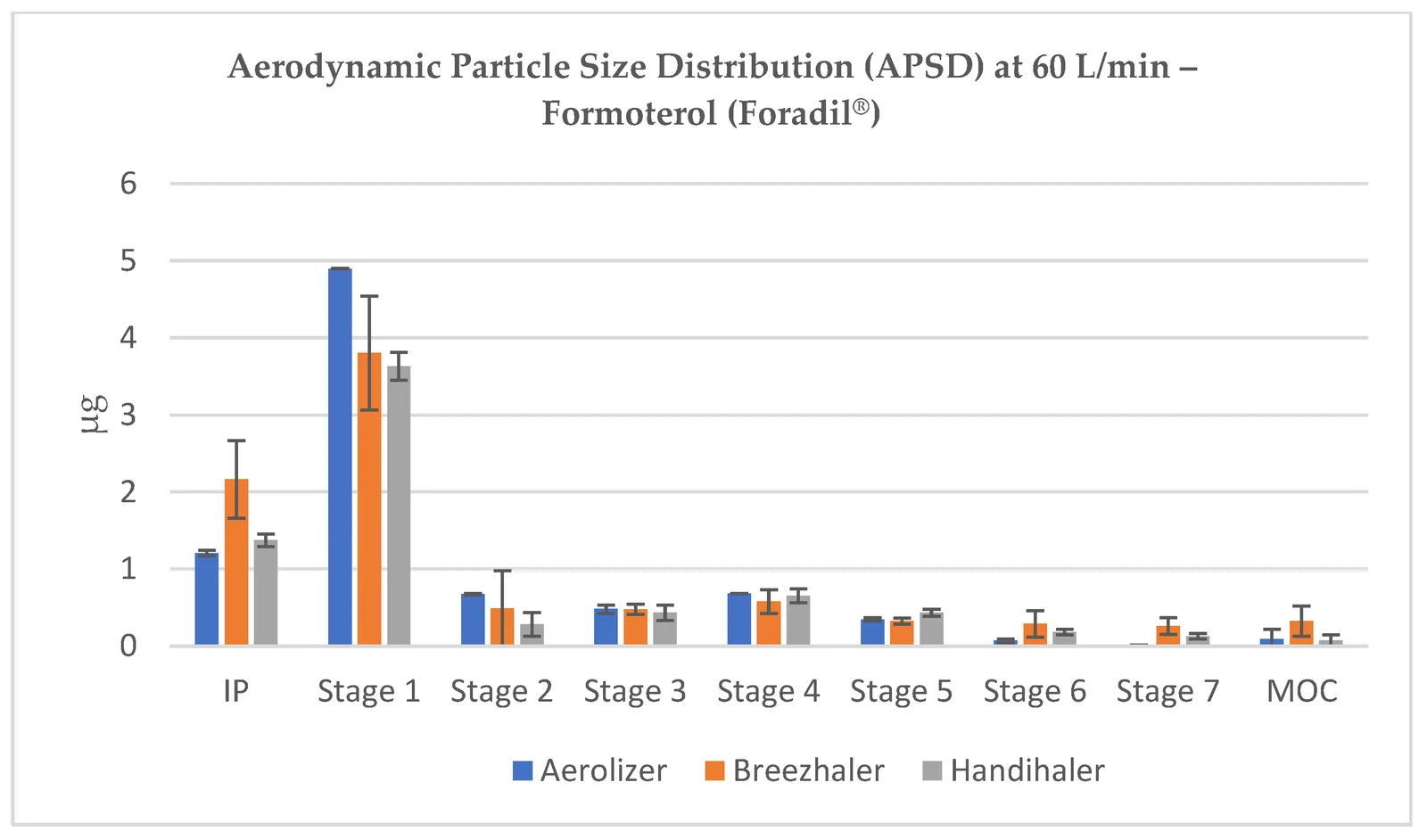
Aerodynamic particle size distribution (APSD) of formoterol from Foradil^®^ capsules at 60 L/min using Aerolizer^®^, Breezhaler^®^, and Handihaler^®^. Mean ± SD (n = 10). One-way ANOVA + Tukey’s HSD for fine particle dose: no significant differences between devices (ns).

At 60 L/min, corresponding to a common inspiratory flow in adults, drug deposition shifted to lower stages in all devices. For Aerolizer and Breezhaler, a notable amount of formoterol appeared in Stage 3 (2.82 µm) and Stage 4 (1.66 µm), indicating the generation of therapeutically relevant particles. Handihaler also showed improved dispersion at this flow, though with visibly reduced deposition on respirable stages (Figure 3).

**Figure 4:**
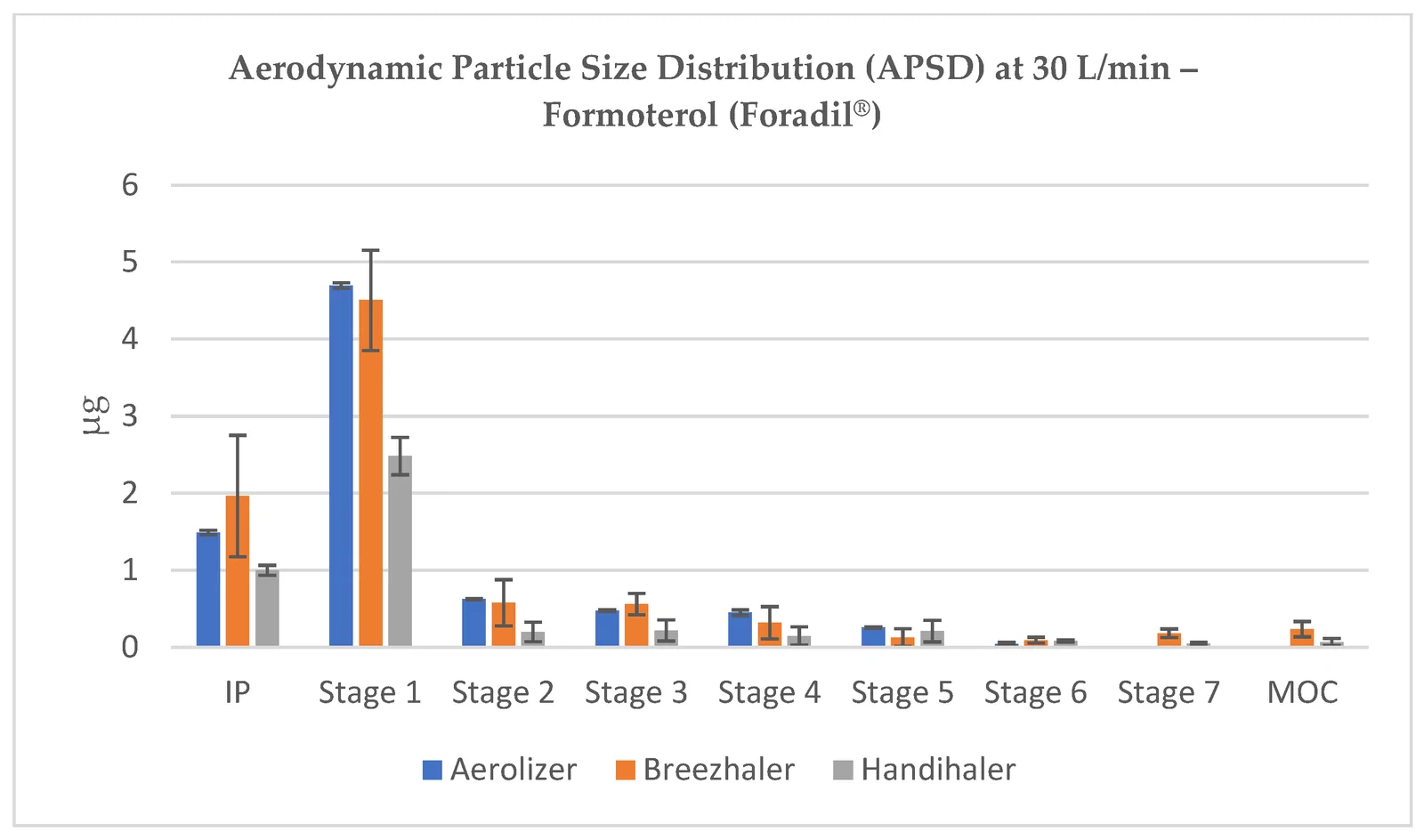
Aerodynamic particle size distribution (APSD) of formoterol from Foradil^®^ capsules at 30 L/min using Aerolizer^®^, Breezhaler^®^, and Handihaler^®^. Mean ± SD (n = 10). One-way ANOVA: *p* = 0.0136. Tukey’s HSD: Breezhaler^®^ vs. Handihaler^®^ *p* < 0.05; all other comparisons not significant (ns).

**Figure 5:**
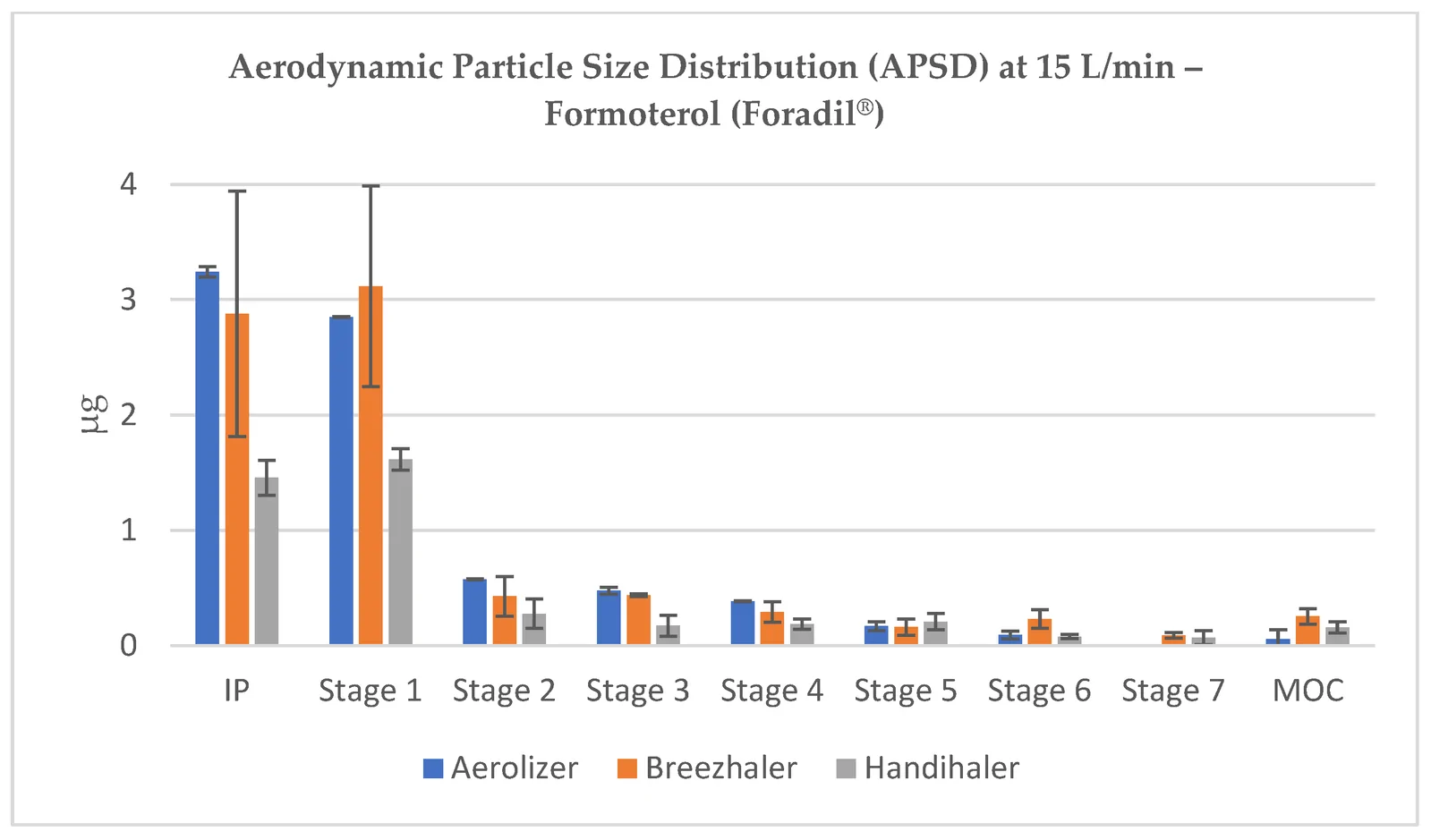
Aerodynamic particle size distribution (APSD) of formoterol from Foradil^®^ capsules at 15 L/min using Aerolizer^®^, Breezhaler^®^, and Handihaler^®^. Mean ± SD (n = 10). One-way ANOVA: *p* < 0.05. Tukey’s HSD: Breezhaler^®^ vs. Handihaler^®^ *p* < 0.05; all other comparisons not significant (ns).

At lower flow rates (15–30 L/min), a substantial portion of the emitted dose was retained in the induction port (IP) and Stage 1, corresponding to cut-off diameters well above 5 µm (e.g., 11.72 and 6.40 µm at 30 L/min). For all devices, the majority of the dose remained in non-respirable regions at these low flow rates, although Breezhaler and Aerolizer deposited larger fractions in Stages 3–5, suggesting relatively better de-agglomeration than Handihaler (Figure 4 and Figure 5).

**Figure 6:**
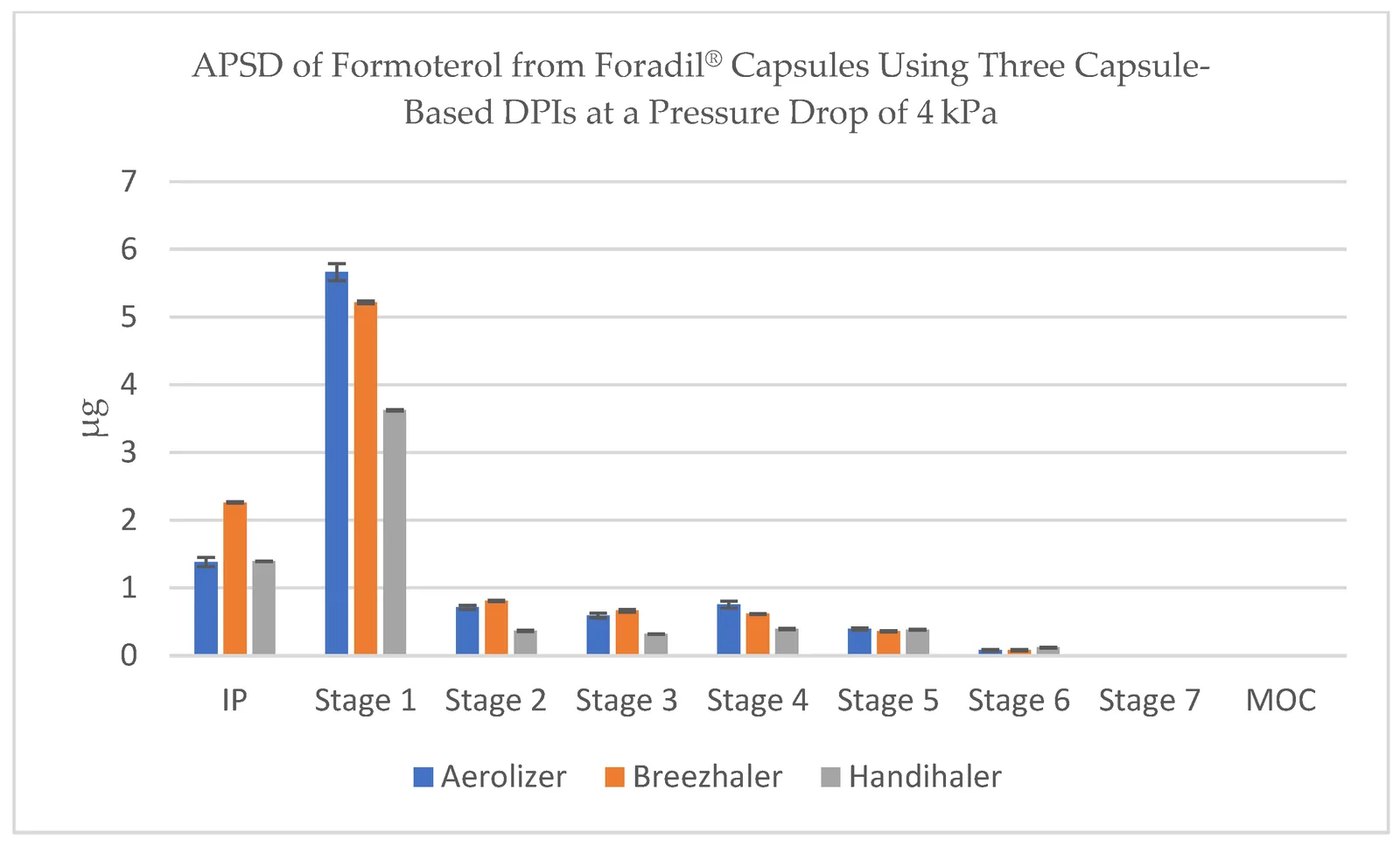
Aerodynamic particle size distribution (APSD) of formoterol from Foradil^®^ capsules at a flow rate corresponding to 4 kPa using Aerolizer^®^, Breezhaler^®^, and Handihaler^®^. Mean ± SD (n = 10). One-way ANOVA: *p* < 0.001. Tukey’s HSD: all pairwise comparisons *p* < 0.001.

When tested at the 4 kPa condition, Breezhaler and Aerolizer continued to show broad distribution across Stages 2–5, consistent with effective de-agglomeration. Conversely, Handihaler produced steep front-loaded deposition, with the majority of the mass confined to IP and Stage 1 (cut-off ~8.78 µm and 4.85 µm, respectively), and minimal transfer to lower stages (Figure 6). This strongly aligned with the reduced FPD measured under this condition.

These results underline the combined influence of inhaler resistance, flow dynamics, and internal geometry on the deposition profile of formoterol-containing powders. The APSD data, aligned with FPD measurements, confirm that Aerolizer and Breezhaler consistently generate a higher proportion of particles in the respirable range, particularly under flow conditions achievable by patients with moderate inspiratory effort.

### 3.3. Influence of Flow Rate on the Fine Particle Dose (FPD) of Glycopyrronium

At 4 kPa, Breezhaler and Aerolizer demonstrated almost equivalent FPD values, reaching 22.05 ± 0.33% and 21.58 ± 0.23%, respectively. In contrast, Handihaler yielded a significantly lower FPD of 10.88 ± 0.13%, underscoring its limited ability to produce respirable particles under optimal pressure-drop conditions. These findings align with the known performance characteristics of low-resistance devices such as Breezhaler and Aerolizer, which are specifically engineered to enhance turbulent de-agglomeration and powder dispersion.

**Figure 7:**
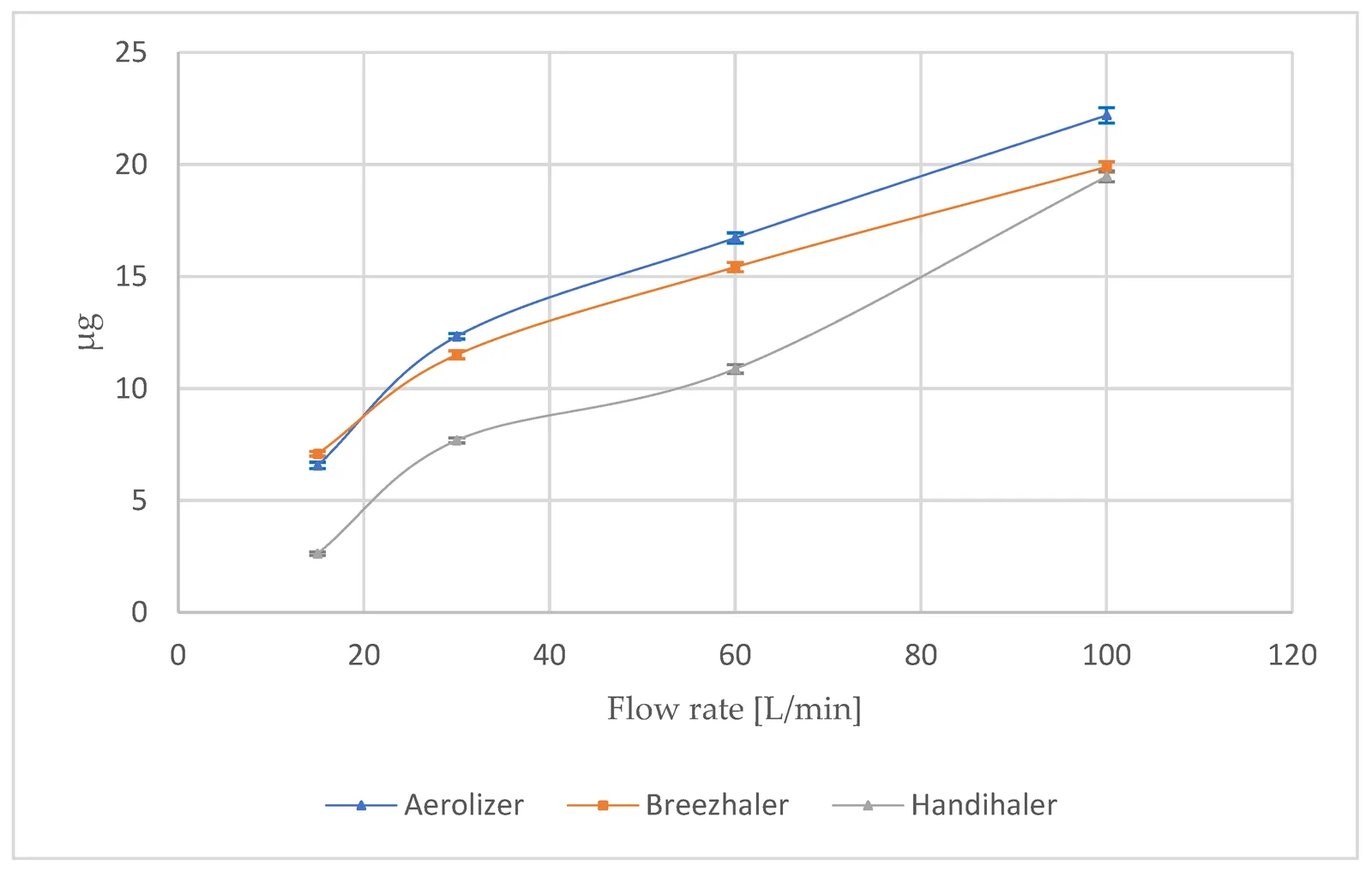
Fine particle dose (FPD, <5 µm) of glycopyrronium emitted from Seebri^®^ capsules using Aerolizer^®^, Breezhaler^®^, and Handihaler^®^ at different flow rates.

Across varying flow rates, a clear trend of decreasing FPD with decreasing inspiratory effort was observed in all inhalers (Figure 7). At 100 L/min, Aerolizer reached 22.21 ± 0.34%, slightly outperforming Breezhaler (19.90 ± 0.23%) and Handihaler (19.47 ± 0.24%). With decreasing flow, the FPD values declined gradually for Aerolizer (16.73 ± 0.23% at 60 L/min, 12.33 ± 0.12% at 30 L/min, 6.57 ± 0.14% at 15 L/min), and similar profiles were observed for Breezhaler (15.43 ± 0.21%, 11.51 ± 0.18%, 7.09 ± 0.10%, respectively). The performance of Handihaler declined more rapidly, falling to 10.88 ± 0.20% at 60 L/min, 7.69 ± 0.11% at 30 L/min, and 3.34 ± 0.08% at 15 L/min.

These data highlight the superior flow-independent performance of Aerolizer and Breezhaler compared to Handihaler. From a clinical perspective, higher FPD values are directly associated with improved lower airway deposition, greater local efficacy, and reduced systemic or oropharyngeal side effects. Devices that sustain high FPD across a range of flow rates provide greater assurance of therapeutic delivery, particularly in patients with impaired inspiratory capacity (e.g., elderly or COPD patients). Based on the data presented in Table 4, the delivered dose (DD) of glycopyrronium was consistently higher for Breezhaler and Aerolizer compared to Handihaler across most flow rates, with Breezhaler showing the greatest DD at the pressure drop of 4 kPa.

### 3.4. Aerodynamic Particle Size Distribution (APSD) of Glycopyrronium

**Figure 8:**
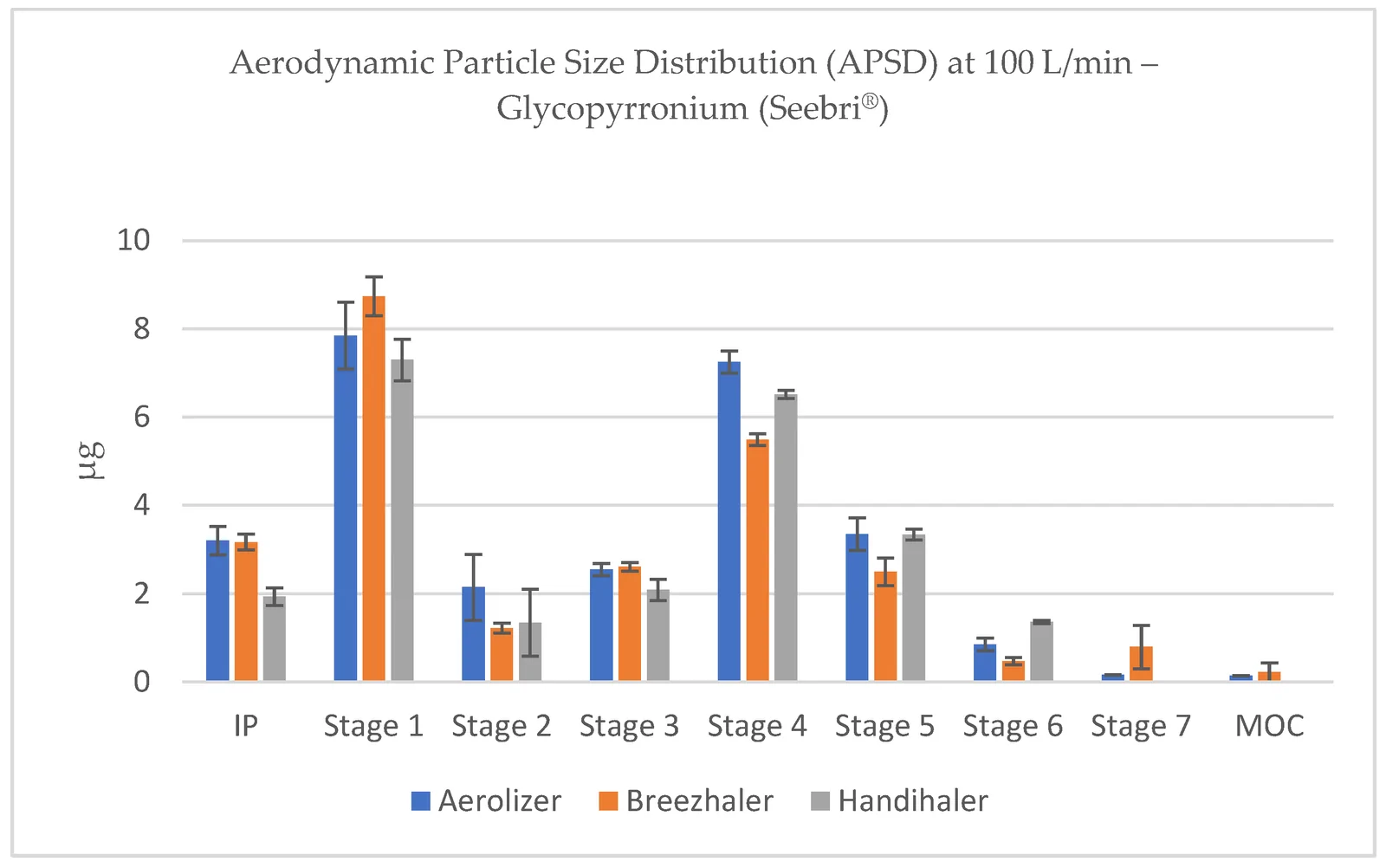
Aerodynamic particle size distribution (APSD) of glycopyrronium from Seebri^®^ capsules at 100 L/min using Aerolizer^®^, Breezhaler^®^, and Handihaler^®^. Mean ± SD (n = 10). One-way ANOVA: *p* < 0.01. Tukey’s HSD: Aerolizer^®^ vs. Breezhaler^®^ *p* < 0.01; Aerolizer^®^ vs. Handihaler^®^ *p* < 0.01; Breezhaler^®^ vs. Handihaler^®^ not significant (ns).

**Figure 9:**
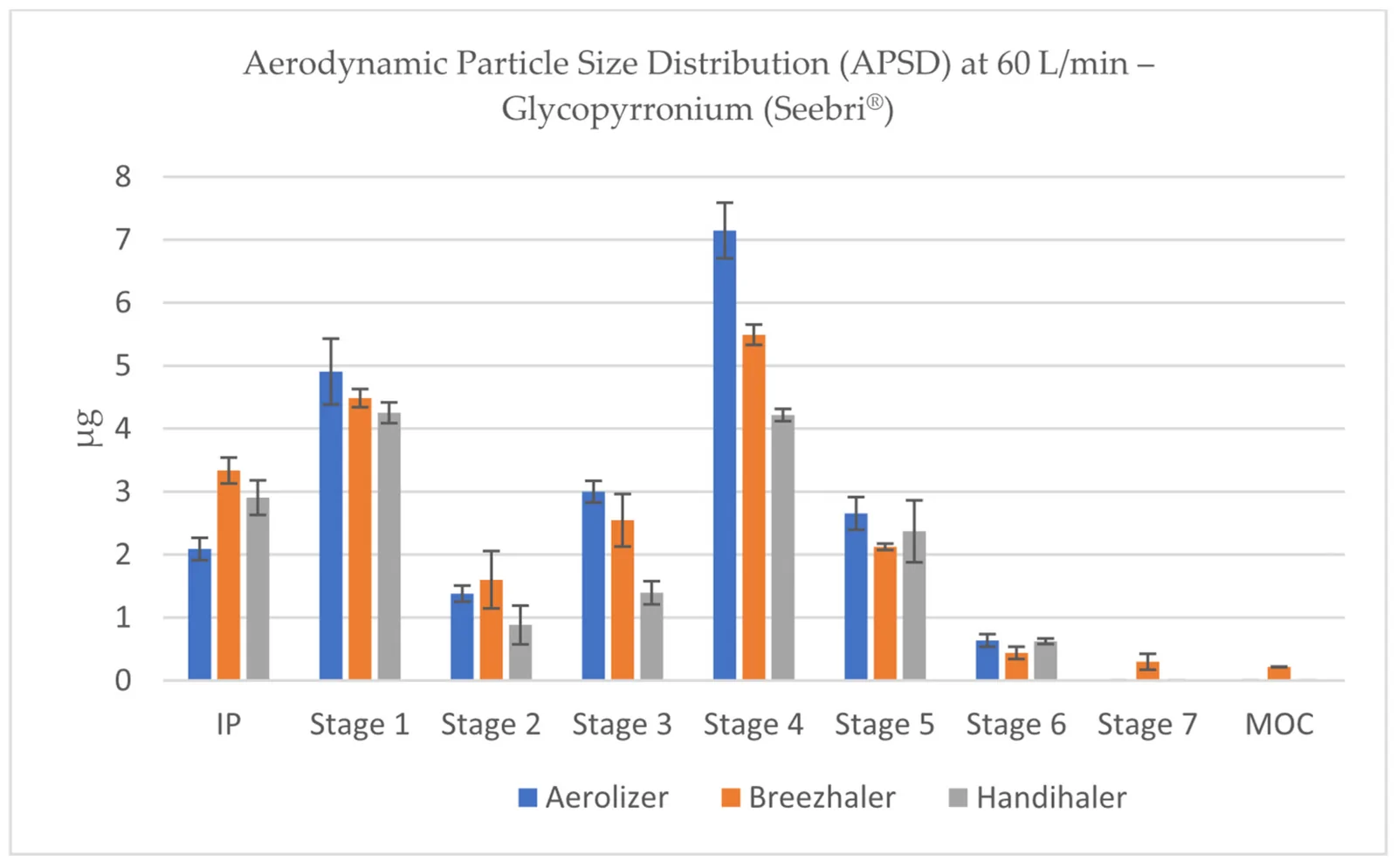
Aerodynamic particle size distribution (APSD) of glycopyrronium from Seebri^®^ capsules at 60 L/min using Aerolizer^®^, Breezhaler^®^, and Handihaler^®^. Mean ± SD (n = 10). One-way ANOVA: *p* < 0.001. Tukey’s HSD: Aerolizer^®^ vs. Handihaler^®^ *p* < 0.001; Breezhaler^®^ vs. Handihaler^®^ *p* < 0.001; Aerolizer^®^ vs. Breezhaler^®^ not significant (ns).

APSD profiles of glycopyrronium aerosols, obtained using the NGI, revealed characteristic deposition patterns for each inhaler under different flow conditions. The stage-by-stage deposition data demonstrated that Aerolizer and Breezhaler consistently produced a major portion of the emitted dose within Stages 2 to 5, which correspond to the respirable fraction of particles below 5 µm (according to flow-specific stage cut-offs). These stages were most prominently represented at 60 and 100 L/min, confirming effective powder dispersion under moderate to high inspiratory flows (Figure 8 and Figure 9).

As inspiratory flow decreased, all devices exhibited a gradual shift in deposition toward earlier stages, but the shift was most pronounced for Handihaler. At 15 L/min, this device demonstrated dominant deposition in the induction port and Stage 1, with minimal mass reaching the deeper NGI stages, indicating insufficient de-agglomeration and suboptimal particle size reduction.

**Figure 10:**
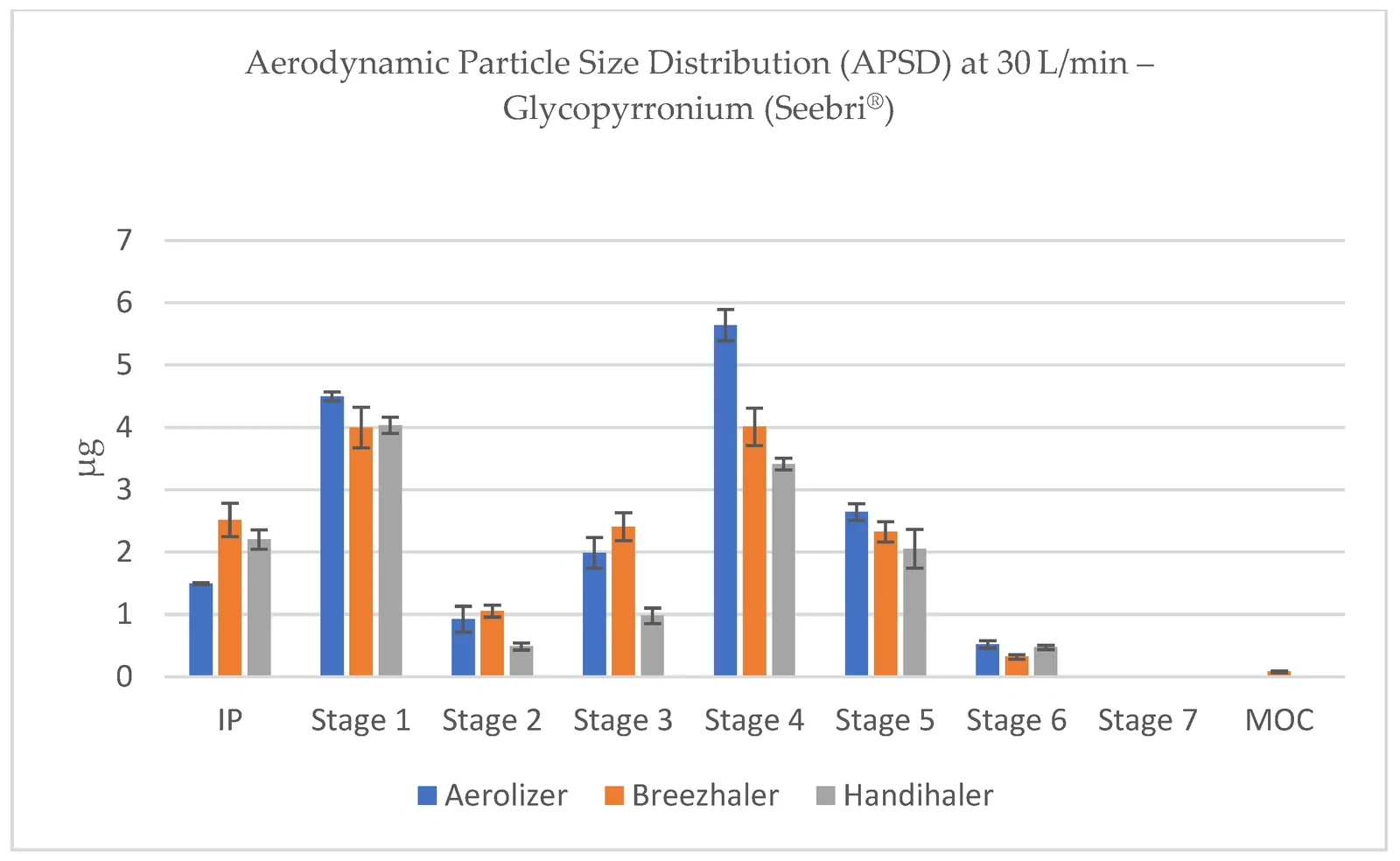
Aerodynamic particle size distribution (APSD) of glycopyrronium from Seebri^®^ capsules at 30 L/min using Aerolizer^®^, Breezhaler^®^, and Handihaler^®^. Mean ± SD (n = 10). One-way ANOVA: *p* < 0.001. Tukey’s HSD: Aerolizer^®^ vs. Handihaler^®^ *p* < 0.001; Breezhaler^®^ vs. Handihaler^®^ *p* < 0.001; Aerolizer^®^ vs. Breezhaler^®^ not significant (ns).

**Figure 11:**
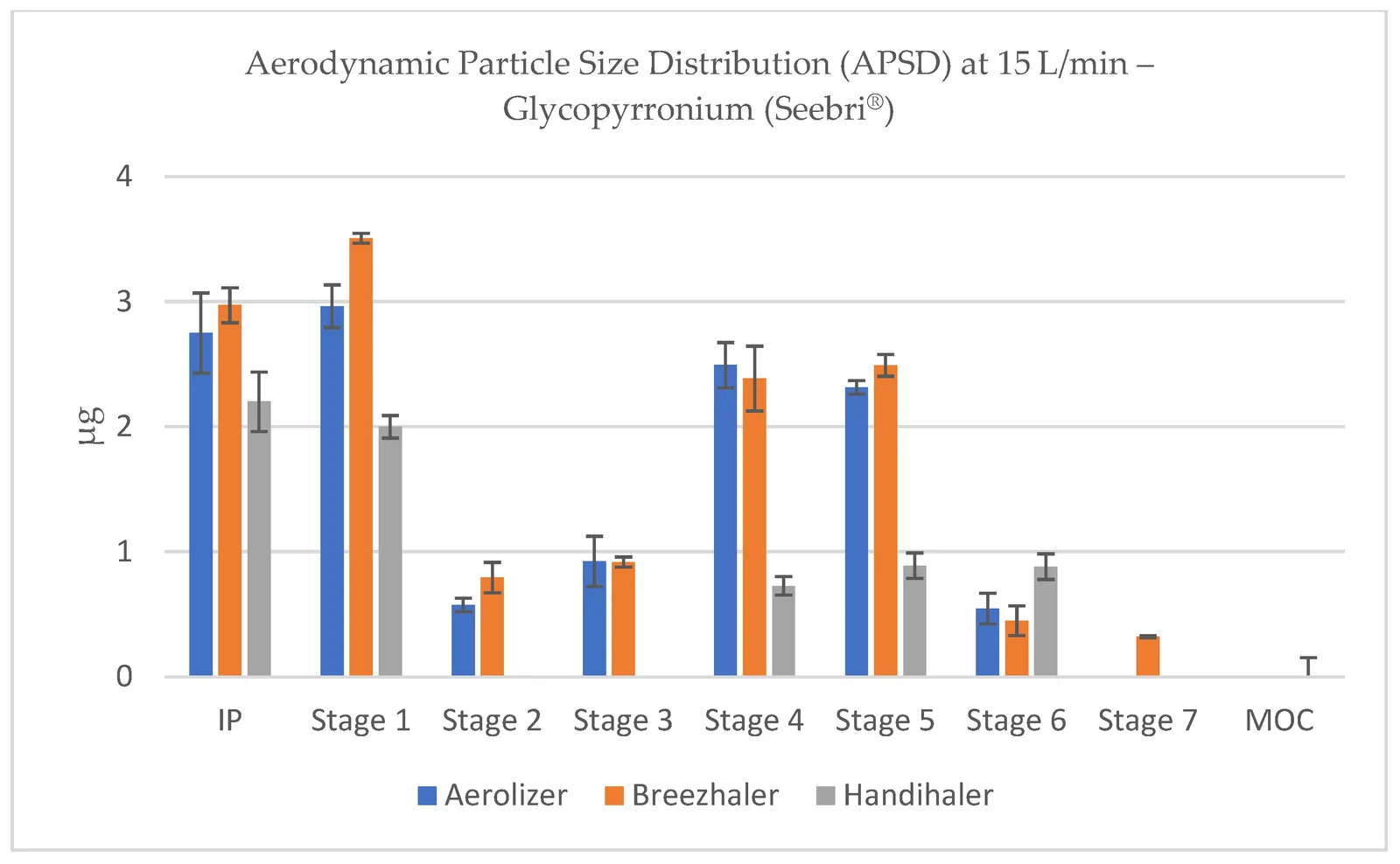
Aerodynamic particle size distribution (APSD) of glycopyrronium from Seebri^®^ capsules at 15 L/min using Aerolizer^®^, Breezhaler^®^, and Handihaler^®^. Mean ± SD (n = 10). One-way ANOVA: *p* < 0.001. Tukey’s HSD: Aerolizer^®^ vs. Handihaler^®^ *p* < 0.001; Breezhaler^®^ vs. Handihaler^®^ *p* < 0.001; Aerolizer^®^ vs. Breezhaler^®^ not significant (ns).

By contrast, Aerolizer and Breezhaler maintained measurable deposition within Stages 3–5 even at low flows (30 and 15 L/min), highlighting their relatively better flow independence and consistent aerosolization performance (Figure 10 and Figure 11). Nevertheless, the absolute FPD values at 15 L/min remained subtherapeutic across all devices, emphasizing the importance of sufficient inspiratory effort for effective DPI use.

**Figure 12:**
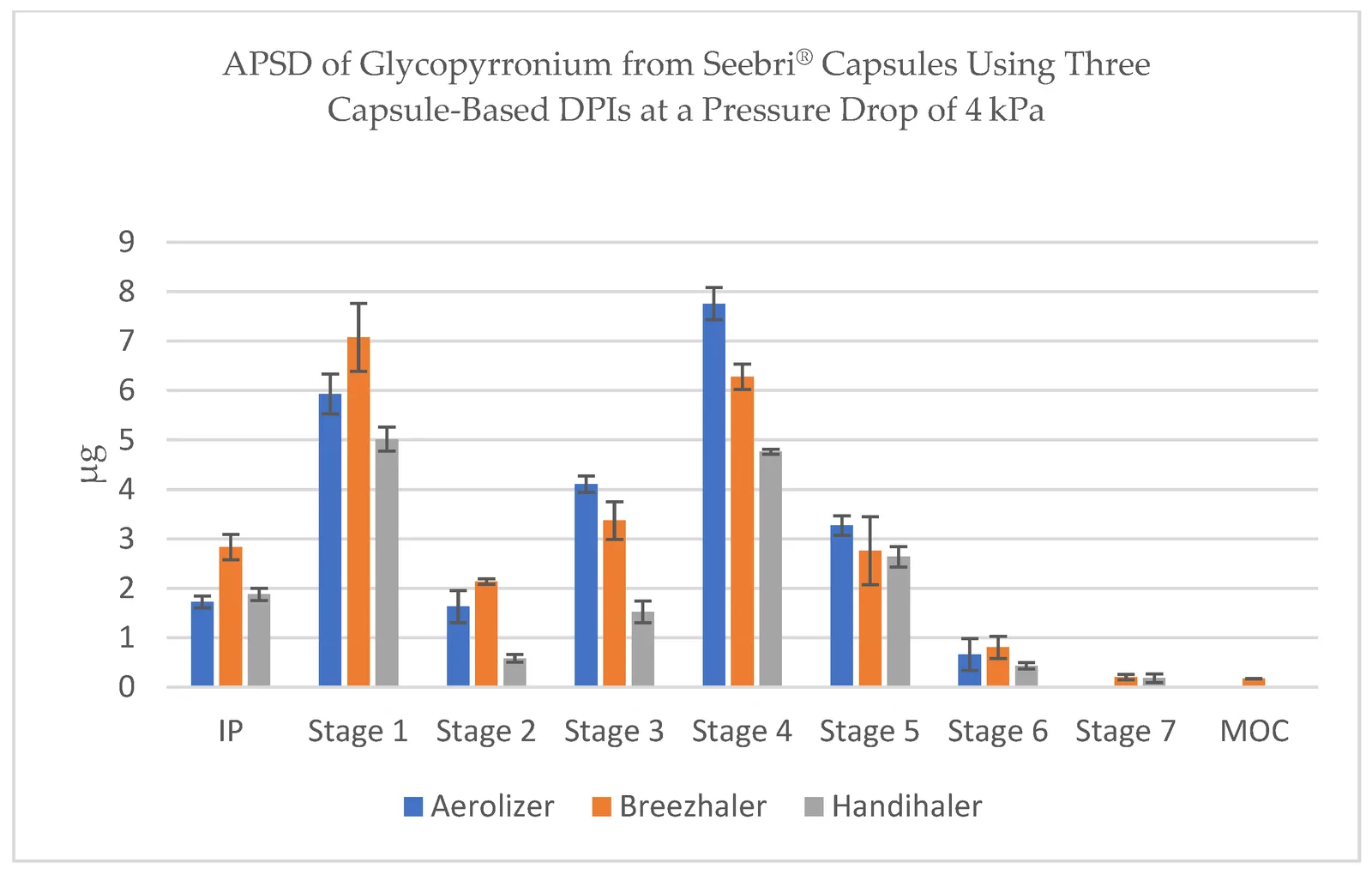
Aerodynamic particle size distribution (APSD) of glycopyrronium from Seebri^®^ capsules at 4 kPa using Aerolizer^®^, Breezhaler^®^, and Handihaler^®^. Mean ± SD (n = 10). One-way ANOVA: *p* < 0.001. Tukey’s HSD: all pairwise comparisons *p* < 0.001.

At 4 kPa, deposition patterns were similar between Aerolizer and Breezhaler, with a mass peak located in Stages 2–4, reflecting optimal aerodynamic particle generation. In contrast, Handihaler showed considerable deposition in Stage 1 and the induction port, indicating a higher proportion of larger particles (>5 µm), and thus a tendency toward oropharyngeal deposition, which may reduce pulmonary efficacy and increase the risk of local side effects (Figure 12).

From a pharmacokinetic and therapeutic standpoint, the APSD profiles reinforce the necessity of matching inhaler resistance and formulation to the target patient population. Devices capable of generating narrow, respirable APSD profiles under real-world inspiratory efforts are more likely to ensure consistent and predictable pulmonary drug delivery, particularly for bronchodilators such as glycopyrronium.

### 3.5. Influence of Flow Rate on the Fine Particle Dose (FPD) of Tiotropium

The highest FPD values were observed under pressure drop-adjusted conditions (4 kPa), for Aerolizer, Breezhaler, and Handihaler, yielding FPD values of 7.85 ± 0.16%, 8.24 ± 0.11%, and 3.30 ± 0.07%, respectively. Under these optimal resistance-compensated settings, both Aerolizer and Breezhaler enabled efficient aerosolization of tiotropium with a comparable fraction of respirable particles, whereas Handihaler displayed noticeably lower dispersibility.

**Figure 13:**
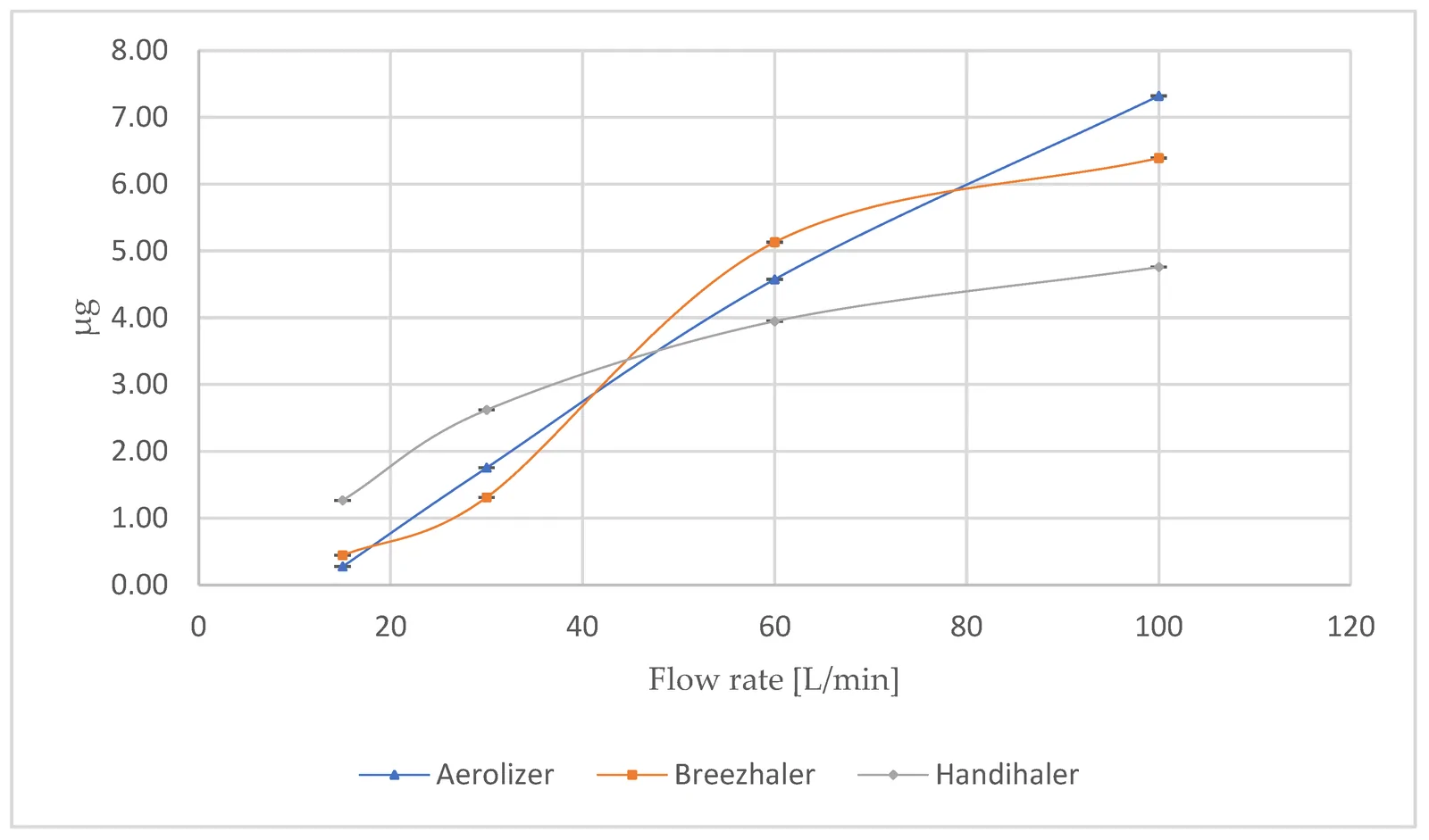
Fine particle dose (<5 µm) of tiotropium emitted from Spiriva^®^ capsules using Aerolizer^®^, Breezhaler^®^, and Handihaler^®^ at different flow rates.

At a fixed flow rate of 100 L/min, FPD values slightly decreased for all devices: 7.32 ± 0.10% for Aerolizer, 6.39 ± 0.00% for Breezhaler, and 4.76 ± 0.09% for Handihaler. A progressive decline in FPD was observed with decreasing flow rates. At 60 L/min, FPDs ranged from 4.58 ± 0.09% (Aerolizer) to 5.13 ± 0.09% (Breezhaler) and 3.95 ± 0.06% (Handihaler). Notably, Breezhaler showed relatively stable performance across a wide range of flow rates, indicating a robust de-aggregation mechanism. Conversely, at very low inspiratory flows (30 and 15 L/min), all devices demonstrated substantially diminished FPDs, falling to ≤2.62% for Handihaler and as low as 0.28% for Aerolizer (Figure 13).

These results indicate that while Breezhaler and Aerolizer offer comparable efficiency at high and moderate flow rates, their performance declines significantly at suboptimal inspiratory efforts. In contrast, Handihaler, despite its moderate-to-high intrinsic resistance, showed a marginally better performance than Aerolizer at the lowest flow (15 L/min), potentially due to longer residence time or reduced capsule motion.

The delivered dose (DD) of tiotropium was highest for Aerolizer at high and moderate flows (Table 5), whereas Breezhaler achieved the greatest DD under a pressure drop of 4 kPa, highlighting device-specific differences in dose emission efficiency.

### 3.6. Aerodynamic Particle Size Distribution (APSD) of Tiotropium

**Figure 14:**
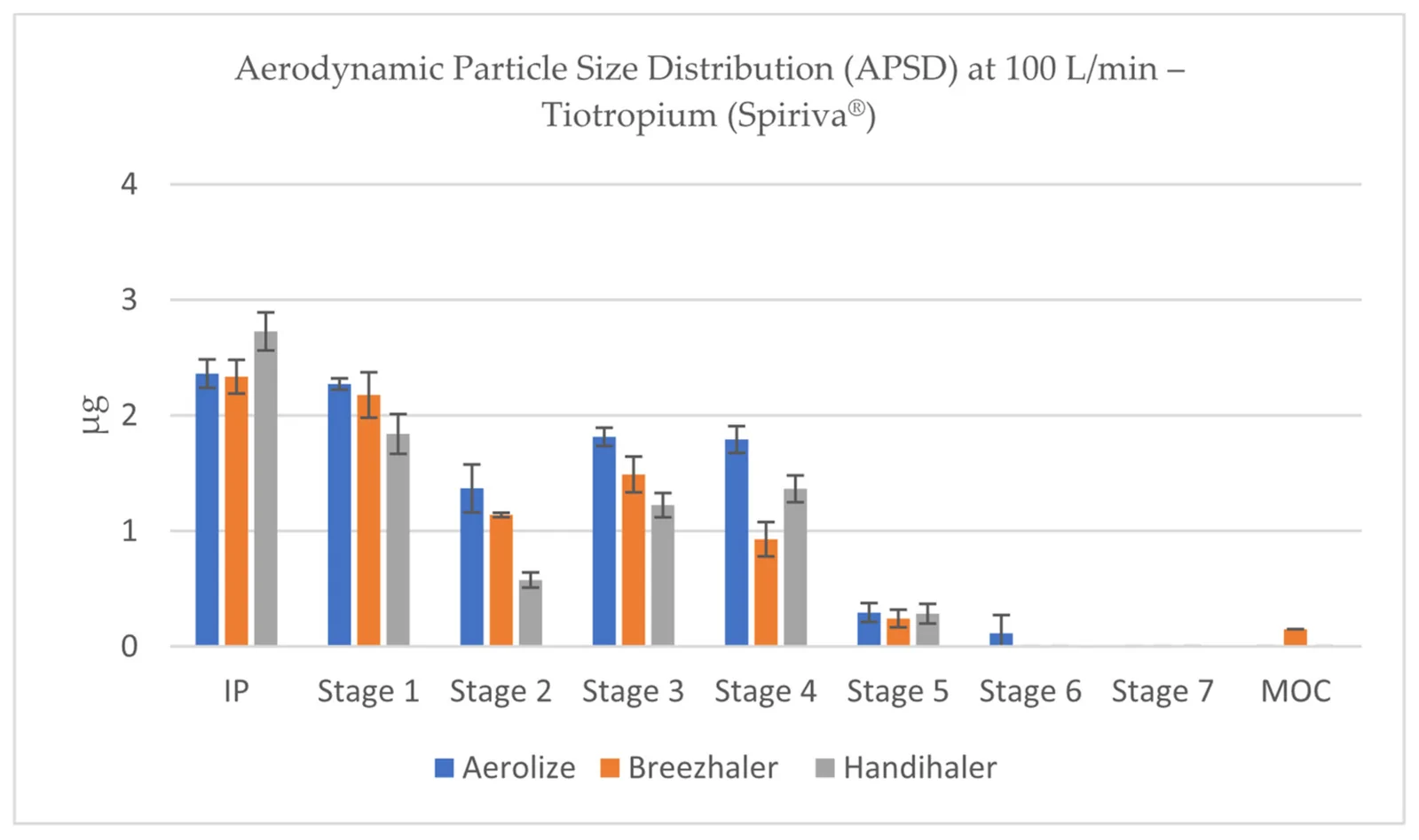
Aerodynamic particle size distribution (APSD) of tiotropium emitted from Spiriva^®^ capsules using Aerolizer^®^, Breezhaler^®^, and Handihaler^®^ at 100 L/min. Data are presented as mean ± SD (n = 10). One-way ANOVA revealed a significant effect of inhaler type (*p* < 0.001); all pairwise comparisons in Tukey’s HSD test were statistically significant (*p* < 0.001).

Analysis of aerodynamic particle size distribution (APSD) profiles across inhalers and flow conditions revealed marked differences in deposition patterns. In contrast, the Handihaler showed a distinct deposition profile with greater accumulation of drug mass in IP and Stage 1 fractions, corresponding to aerodynamic diameters >5 µm (Figure 14). This shift toward coarser aerosol fractions is consistent with the lower FPD values and suggests significant upper airway and oropharyngeal deposition when Handihaler is used to deliver tiotropium, especially under low-flow conditions.

**Figure 15:**
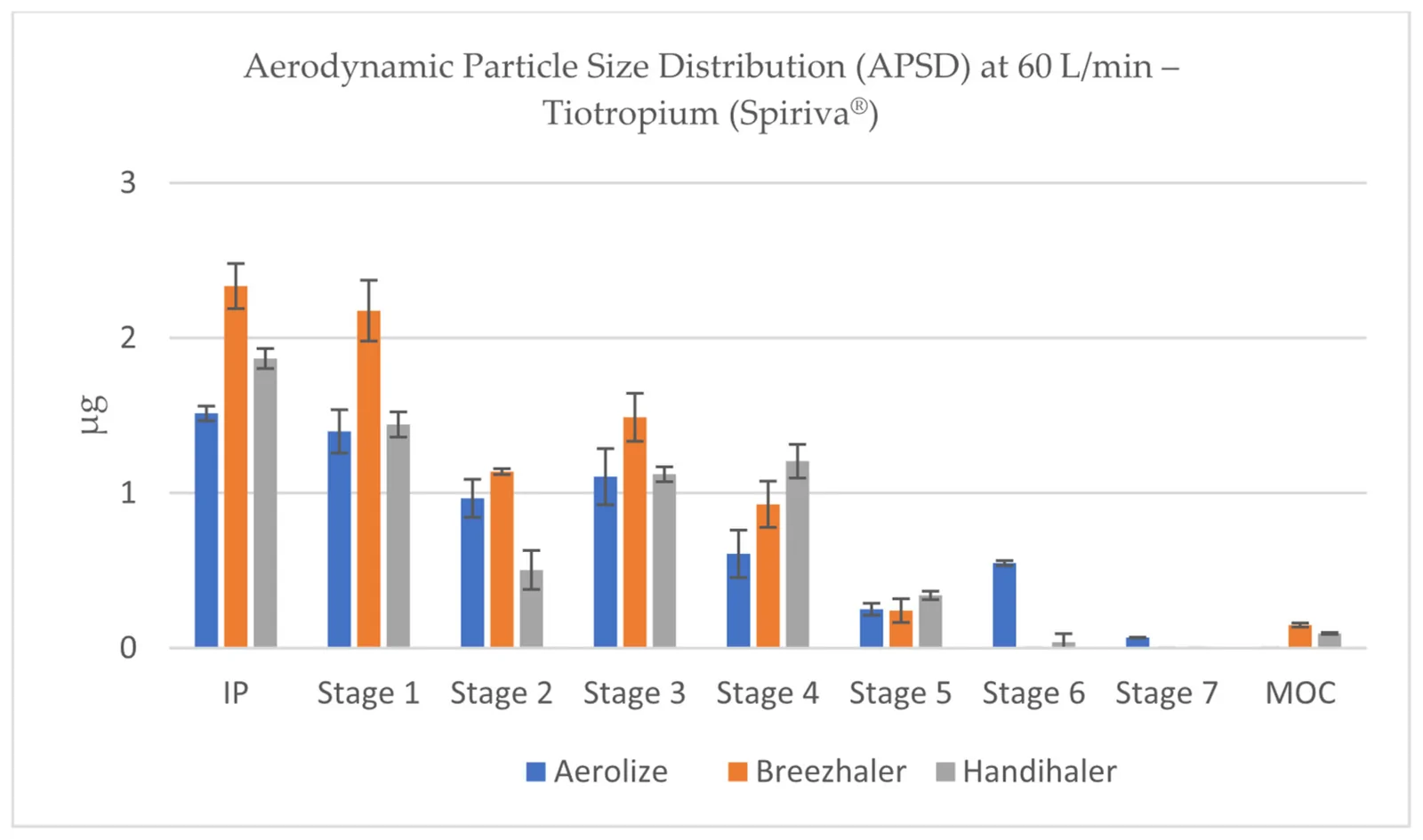
Fine particle dose (<5 µm) of tiotropium emitted from Spiriva^®^ capsules using Aerolizer^®^, Breezhaler^®^, and Handihaler^®^ at 60 L/min. Data are presented as mean ± SD (n = 10). One-way ANOVA showed a significant effect of inhaler type (*p* < 0.05). Tukey’s HSD test revealed significant differences for Breezhaler^®^ vs. Handihaler^®^ (*p* < 0.01), while other pairwise comparisons were not statistically significant.

**Figure 16:**
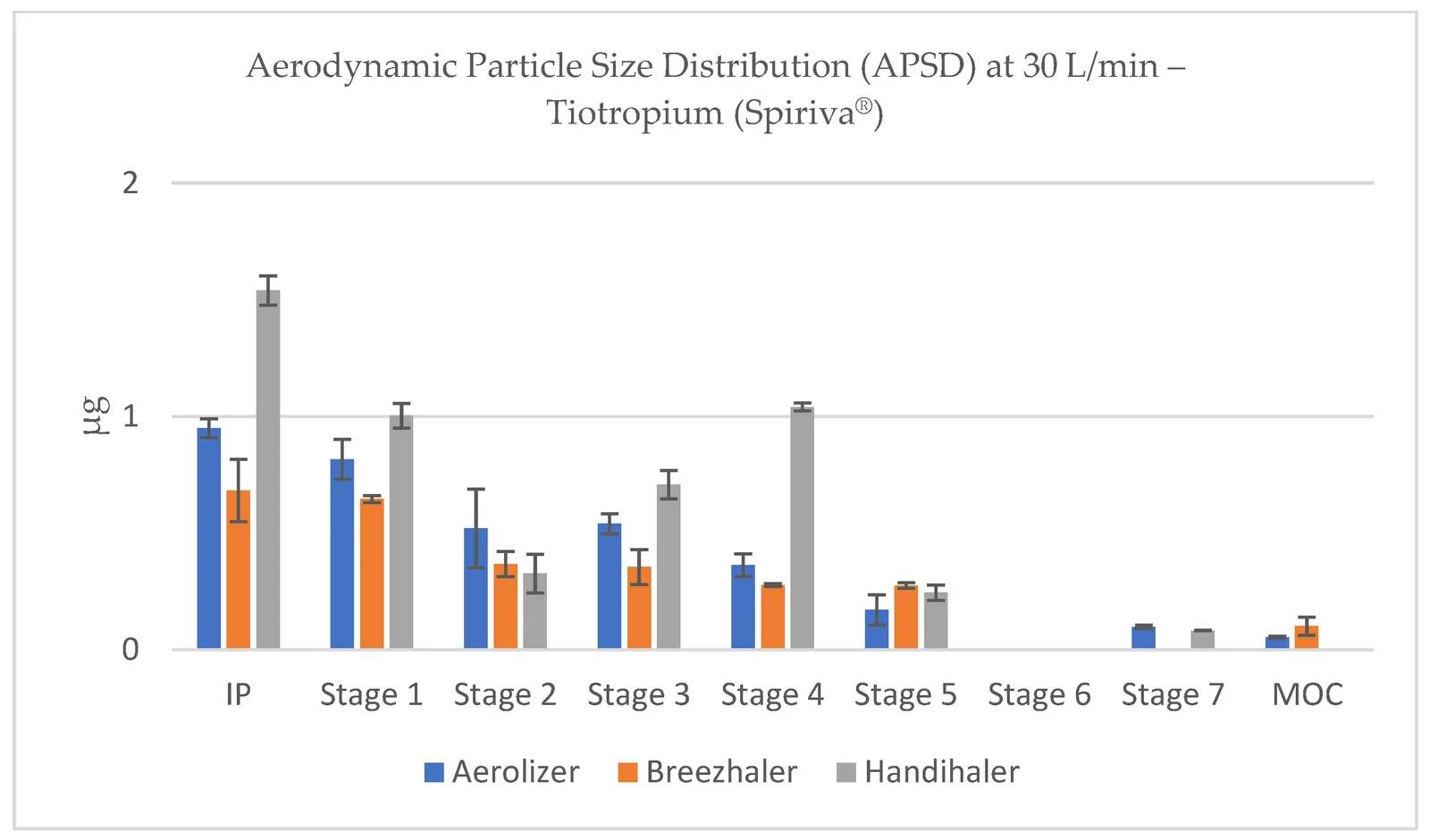
Fine particle dose (<5 µm) of tiotropium emitted from Spiriva^®^ capsules using Aerolizer^®^, Breezhaler^®^, and Handihaler^®^ at 30 L/min. Data are shown as mean ± SD (n = 10). One-way ANOVA indicated a significant effect of inhaler type (*p* < 0.01). Tukey’s HSD test showed significantly higher FPD values for Aerolizer^®^ vs. Handihaler^®^ (*p* < 0.01) and Breezhaler^®^ vs. Handihaler^®^ (*p* < 0.05); differences between Aerolizer^®^ and Breezhaler^®^ were not statistically significant.

**Figure 17:**
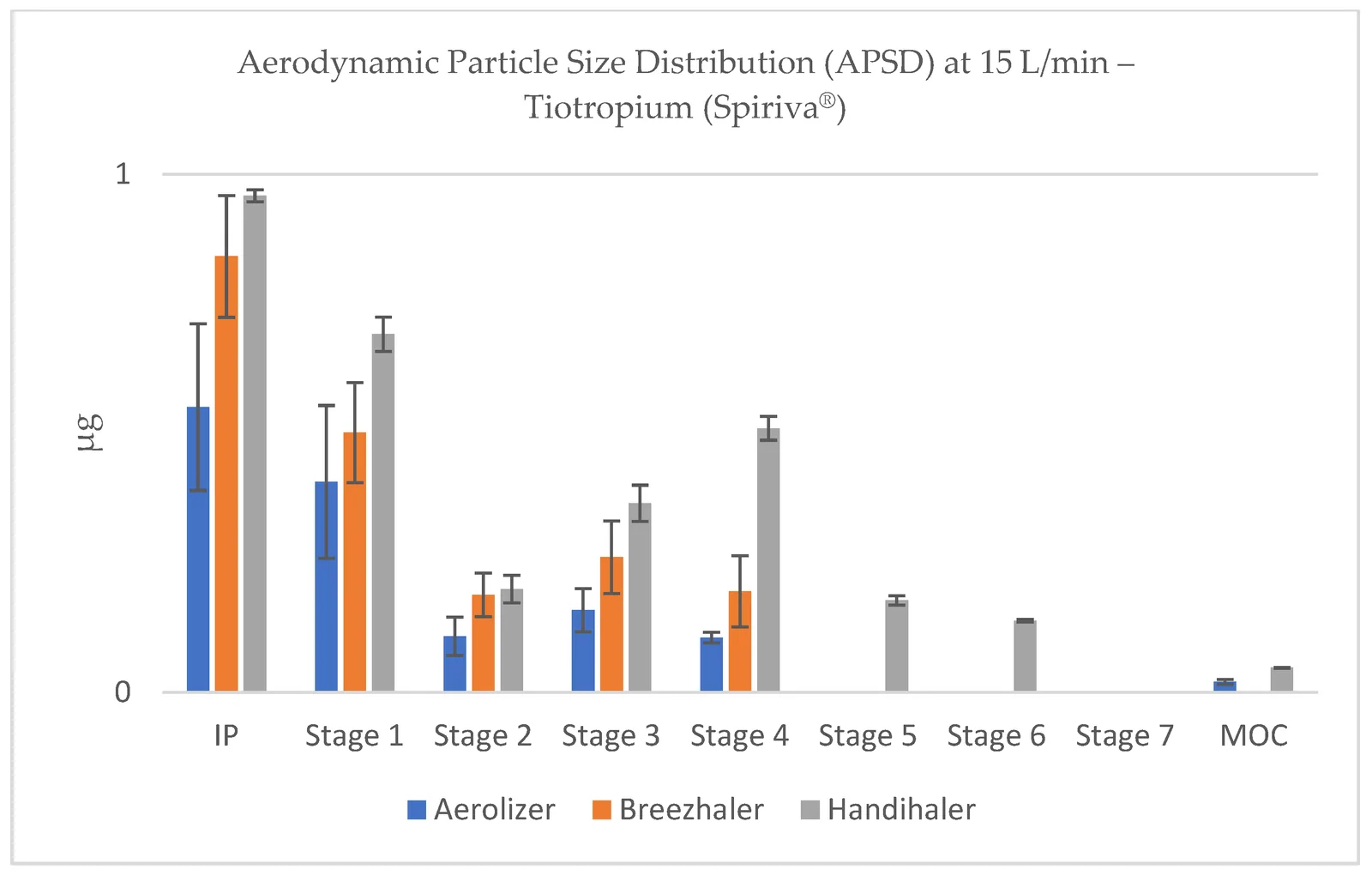
Fine particle dose (<5 µm) of tiotropium emitted from Spiriva^®^ capsules using Aerolizer^®^, Breezhaler^®^, and Handihaler^®^ at 15 L/min. Data are presented as mean ± SD (n = 10). One-way ANOVA indicated a significant effect of inhaler type (*p* < 0.001). Tukey’s HSD test showed significantly higher FPD values for Aerolizer^®^ vs. Handihaler^®^ (*p* < 0.05), Breezhaler^®^ vs. Handihaler^®^ (*p* < 0.001), and Aerolizer^®^ vs. Breezhaler^®^ (*p* < 0.05).

At reduced flow rates (60–30 L/min), both Aerolizer and Breezhaler showed a gradual leftward shift in deposition (toward larger particles), with a relative increase in drug mass recovered in Stages 1–2 and decreased deposition in Stages 4–6 (Figure 15 and Figure 16). This pattern indicates less effective powder de-agglomeration and slower acceleration of particles due to reduced shear forces. At the lowest flow tested (15 L/min), particle size distributions became markedly coarse for all devices, particularly for Handihaler, where most deposition occurred in IP and Stage 1, confirming the device’s strong dependency on adequate inspiratory effort (Figure 17).

**Figure 18:**
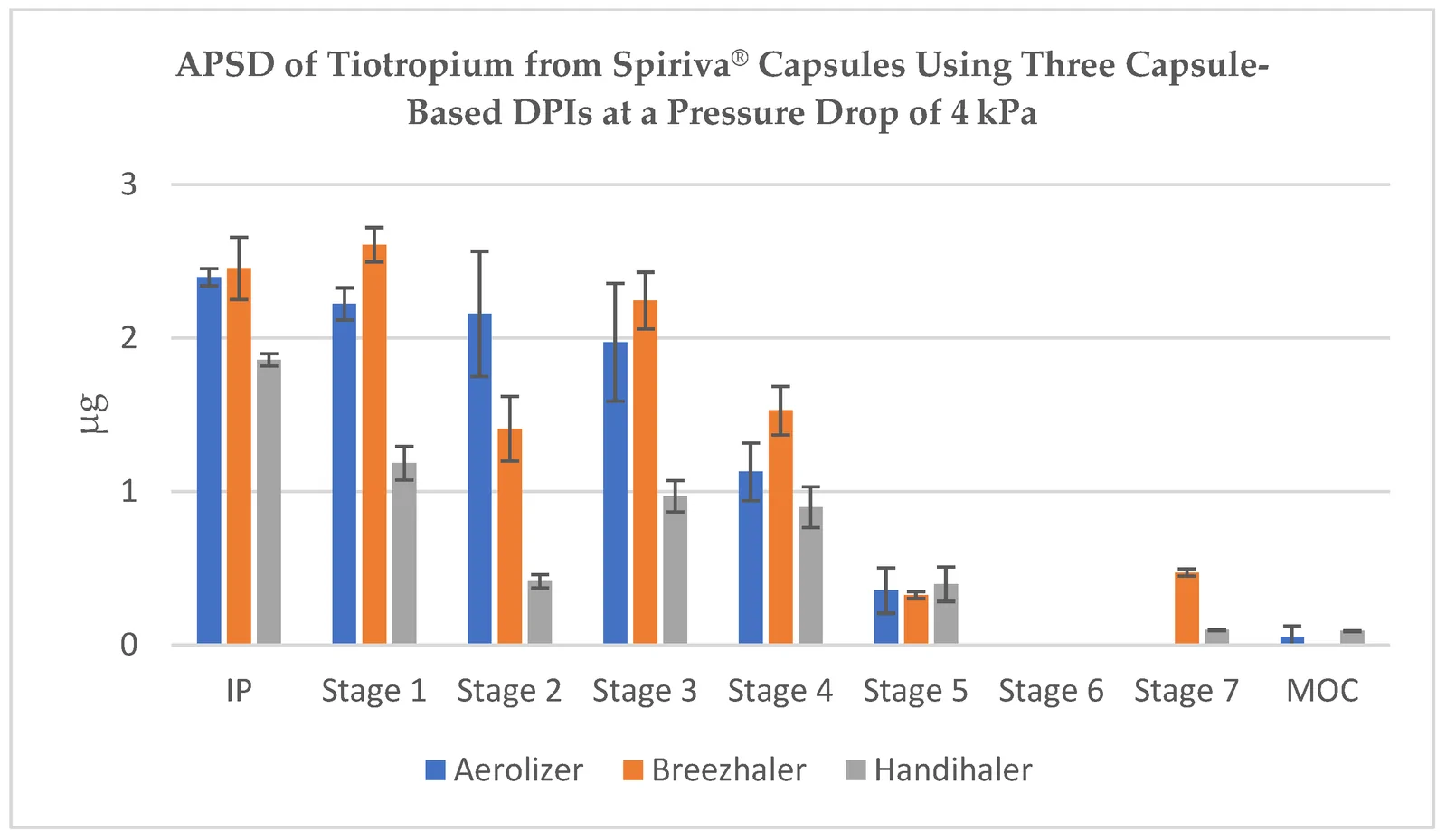
Fine particle dose (<5 µm) of tiotropium emitted from Spiriva^®^ capsules using Aerolizer^®^, Breezhaler^®^, and Handihaler^®^ at 4 kPa. Data are presented as mean ± SD (n = 10). One-way ANOVA revealed a significant effect of inhaler type (*p* < 0.001). Tukey’s HSD test indicated significantly higher FPD values for Breezhaler^®^ vs. Handihaler^®^ (*p* < 0.001) and Aerolizer^®^ vs. Handihaler^®^ (*p* < 0.001), whereas the difference between Aerolizer^®^ and Breezhaler^®^ was not statistically significant (*p* > 0.05).

At 4 kPa, Breezhaler and Aerolizer generated the highest quantities of particles within the respirable size range (<5 µm), with mass deposition peaks occurring predominantly in Stages 2–4 of the NGI, corresponding to aerodynamic cut-offs between ~3.3 and 1.3 µm (depending on device-specific flow rate). These stages consistently accounted for the largest portion of total recovered dose for both devices, reflecting efficient de-agglomeration and dispersion (Figure 18).

Overall, the APSD profiles support the FPD findings and underline the importance of inhalation strength and device selection for effective pulmonary delivery of tiotropium. These results are particularly relevant for patient populations with compromised inspiratory capacity, such as elderly or COPD patients.

### 3.7. Statistical Comparison of Fine Particle Dose (FPD) Across Devices and Flow Rates

To assess the statistical significance of differences in Fine Particle Dose (FPD) among the three tested inhalers—Aerolizer, Breezhaler^®^, and Handihaler^®^—for each of the studied active substances (formoterol, glycopyrronium, and tiotropium), one-way ANOVA followed by Tukey’s HSD post hoc test were performed at each flow rate.

For formoterol (Foradil^®^ capsules), statistically significant differences in FPD were observed across all flow rates (*p* < 0.05). At the lowest flow rate (15 L/min), Breezhaler^®^ delivered significantly more fine particles than Aerolizer and Handihaler^®^, with the latter producing the lowest FPD (*p* < 0.01). As flow increased, the differences narrowed; at 60 L/min and 100 L/min, all three inhalers provided comparable FPD values, though Aerolizer maintained a slight numerical advantage. At the pressure drop of 4 kPa, both Aerolizer and Breezhaler^®^ outperformed Handihaler^®^ (*p* < 0.01), while no significant difference was detected between the two low-resistance devices (*p* > 0.05).

For glycopyrronium (Seebri^®^ capsules), ANOVA confirmed significant variation in FPD between devices at all tested flow rates (*p* < 0.05). Breezhaler^®^ demonstrated the highest FPD at 15 and 30 L/min, significantly surpassing both Aerolizer and Handihaler^®^ (*p* < 0.01). As flow increased, Aerolizer gradually matched and then exceeded Breezhaler^®^ in FPD delivery at 60 L/min and 100 L/min (*p* < 0.05 vs. Handihaler^®^). At 4 kPa, Aerolizer and Breezhaler^®^ again performed significantly better than Handihaler^®^ (*p* < 0.01), with no significant difference between them.

For tiotropium (Spiriva^®^ capsules), the lowest flow rate (15 L/min) revealed significant differences in FPD, with Aerolizer and Breezhaler^®^ yielding superior deposition compared to Handihaler^®^ (*p* < 0.01). This trend persisted at 30 L/min. At higher flow rates (60 and 100 L/min), differences between Aerolizer and Breezhaler^®^ diminished, though both remained significantly more effective than Handihaler^®^ (*p* < 0.05). Under 4 kPa conditions, Aerolizer and Breezhaler^®^ continued to deliver significantly higher FPD than Handihaler^®^ (*p* < 0.01), and no statistical difference was observed between the low-resistance inhalers (*p* > 0.05).

Across all active substances, Handihaler^®^ consistently exhibited lower FPD values at reduced flow rates, underscoring its dependency on higher inspiratory effort. In contrast, Aerolizer and Breezhaler^®^ provided more efficient aerosolization under variable flow conditions, particularly for patients with limited inhalation capacity. These findings align with device design characteristics, including airflow resistance, capsule orientation, and piercing mechanisms, and demonstrate the necessity of matching the inhaler to patient-specific inspiratory profiles.

## 4. Discussion and Conclusions

The observed differences in aerodynamic performance among the tested dry powder inhalers (DPIs) can be attributed to distinct structural and functional characteristics of each device. Several key design parameters—including capsule orientation, piercing mechanism, and intrinsic airflow resistance—affect the fluid dynamics within the inhaler and, consequently, the efficiency of powder de-aggregation and aerosol generation.

Both Aerolizer^®^ and Breezhaler^®^ are low-resistance inhalers, while Handihaler^®^ exhibits medium-to-high resistance, requiring higher inspiratory effort to achieve equivalent pressure drops. Lower-resistance inhalers facilitate rapid airflow with increased turbulence intensity, which enhances capsule motion and powder dispersion at moderate flow rates. In contrast, higher-resistance devices tend to generate more laminar flow under equivalent pressure conditions, resulting in less efficient particle de-aggregation unless substantial inhalation effort is provided by the patient [21].

Capsule orientation is another critical parameter. In both Aerolizer^®^ and Breezhaler^®^, the capsules are held horizontally, allowing for optimal oscillation and rotation during inhalation. In contrast, Handihaler^®^ positions the capsule vertically, which may limit its rotational dynamics and reduce the kinetic energy transfer to the powder payload [22]. Additionally, Aerolizer^®^ employs a system of eight piercing needles, resulting in a greater number of air inlet holes and improved airflow interaction with the capsule interior. In comparison, Breezhaler^®^ and Handihaler^®^ utilize two-needle systems, which may constrain the airflow jet angles and reduce the uniformity of capsule movement [23].

These structural differences are reflected in the aerodynamic particle size distributions (APSDs) and fine particle fractions (FPFs) obtained across devices. Studies utilizing computational fluid dynamics (CFD) modeling have demonstrated that capsule oscillation frequency, airflow patterns, and turbulence levels inside the inhaler chamber directly influence the fragmentation of agglomerates and drug deposition profiles [24,25]. Although no CFD simulations were conducted as part of the current study, our results align with established theoretical and experimental findings in this field. Taken together, these insights underscore the importance of inhaler architecture in determining the in vitro aerosol performance of DPI formulations. The interdependence between device resistance, airflow trajectory, and powder dispersion must be considered when interpreting comparative studies, particularly when evaluating the performance of non-dedicated inhalers with formulations they were not originally designed for.

In this study, a comprehensive in vitro comparison of three capsule-based dry powder inhalers (DPIs)—Aerolizer, Breezhaler, and Handihaler—was conducted using commercially available formulations of formoterol fumarate, glycopyrronium bromide, and tiotropium bromide monohydrate. Aerosolization performance was assessed under a broad range of inspiratory flow rates, including standardized conditions (15, 30, 60, 100 L/min) as well as physiologically relevant pressure drop conditions (~4 kPa). Key parameters, including the fine particle dose (FPD) and aerodynamic particle size distribution (APSD), were quantified using the NGI in accordance with compendial procedures.

Across all tested substances, a marked dependence of aerosolization efficiency on flow rate was observed. As expected, higher inspiratory flow conditions led to improved powder dispersion and increased deposition of the drug within the respirable fraction (<5 µm), as reflected by elevated FPD values. Conversely, at lower flow rates, especially 15 and 30 L/min, reduced de-aggregation of particles resulted in a significant shift of mass toward larger aerodynamic diameters, which correspond to increased deposition in the oropharyngeal cavity and upper airways [26,27].

This trend was particularly evident in the case of formoterol, where the highest FPD was observed at 4 kPa flow conditions using the Aerolizer and Breezhaler devices. In contrast, the Handihaler exhibited limited performance enhancement under elevated flow, likely due to its higher intrinsic resistance and capsule orientation. Glycopyrronium and tiotropium also showed improved respirable dose delivery at higher flows, with the Breezhaler and Aerolizer achieving superior FPDs compared to the Handihaler. Notably, for all three drugs, the relative differences in FPD between devices diminished as flow rate increased, suggesting that sufficient inspiratory effort can, to some extent, compensate for mechanical design differences.

Despite the use of identical aerodynamic assessment methods and comparable testing conditions, notable differences were observed in the deposition profiles of the three tested active pharmaceutical ingredients (APIs): formoterol, glycopyrronium, and tiotropium. Both formoterol and glycopyrronium demonstrated appreciable deposition in NGI Stages S5–S7 across all devices, indicating efficient de-agglomeration and aerosolization into the respirable size range. In contrast, tiotropium exhibited substantially reduced deposition in these same stages, with a greater proportion of mass retained in the upper NGI stages corresponding to larger aerodynamic diameters. This variability can be attributed to the distinct physicochemical properties of each API as well as formulation-specific factors. Glycopyrronium, for example, is formulated in Seebri^®^ capsules with the addition of magnesium stearate—a well-known dispersibility enhancer that reduces cohesive forces between the drug and the lactose carrier, thereby facilitating powder detachment and entrainment. Formoterol, though formulated without magnesium stearate, is also known to possess favorable dispersibility characteristics due to its relatively low hygroscopicity and cohesive behavior. In contrast, tiotropium bromide monohydrate (Spiriva^®^) is formulated without dispersion-enhancing excipients and is recognized for its strong affinity to lactose carriers, which likely hampers effective de-agglomeration, particularly at lower flow rates. Furthermore, the interaction between powder and inhaler airflow dynamics may exacerbate this effect. The observed reduction in fine particle deposition for tiotropium across all devices may therefore be a function of both inherent powder properties and suboptimal detachment kinetics under the applied flow regimes. Taken together, these findings emphasize the critical importance of API-specific formulation strategies in dry powder inhalation therapy. While device architecture plays a significant role, it cannot fully compensate for suboptimal powder dispersibility. Thus, consistent therapeutic performance relies not only on device design but also on the tailored physicochemical and excipient properties of each formulation.

Importantly, clinical implications arise from the observed variability in aerodynamic dispersion. It is well established that particles within the respirable range (<5 µm) are capable of reaching the lower bronchial tree, where they can exert their intended pharmacological effect. Conversely, particles exceeding this aerodynamic threshold are prone to deposit in the mouth–throat region, reducing therapeutic efficacy and increasing the risk of local and systemic side effects due to undesired absorption patterns. Therefore, a higher FPD is generally associated with improved clinical outcomes, reduced dosing frequency, and better safety profiles.

While the data presented herein suggest that, under certain flow conditions, non-dedicated devices (e.g., Aerolizer used with glycopyrronium or tiotropium) may outperform their proprietary counterparts in terms of respirable dose, these findings should be interpreted with caution. Inhalers are developed and registered as integral parts of a drug–device combination product, with their safety and efficacy profiles established through rigorous clinical studies. The substitution of inhalation devices, even with comparable aerodynamic performance, may lead to variability in drug delivery, altered pharmacokinetics, or compromised patient usability, particularly in populations with reduced inspiratory capacity.

Furthermore, inhaler resistance, capsule piercing mechanism, and powder dispersion pathways differ significantly among devices. For instance, the vertical orientation of the capsule in the Handihaler and its dual-needle puncture system contrast with the horizontal capsule placement and distinct needle configurations in the Aerolizer and Breezhaler. These structural variations influence not only airflow patterns but also capsule vibration and powder entrainment dynamics. As such, device-specific formulation characteristics (e.g., lactose grade, capsule material, excipient blend) are optimized to match the aerodynamic and mechanical properties of the inhaler during development. Deviation from this pairing may lead to suboptimal clinical outcomes despite favorable in vitro performance [28]. Nevertheless, from a regulatory and clinical standpoint, it is therefore strongly recommended to adhere to the prescribed inhaler specified for each drug product. Any modifications or substitutions should be approached under strict pharmaceutical and clinical supervision, ideally supported by bridging studies or real-world evidence demonstrating non-inferiority in clinical endpoints.

In summary, this study highlights the substantial impact of both inspiratory flow rate and inhaler design on the aerodynamic performance of capsule-based dry powder inhalers (DPIs). The results underscore the importance of individualized inhaler selection, taking into account not only the drug formulation but also patient-specific inspiratory capacity and device-handling abilities. Although in vitro performance provides valuable insight into expected deposition profiles, it represents only one facet of the broader therapeutic landscape, which must also encompass usability, adherence, and clinical effectiveness. Further innovation in DPI design should focus on enhancing dispersion efficiency at lower flow rates while maintaining intuitive usability, in order to maximize therapeutic benefit across diverse patient populations. Nevertheless, while certain non-dedicated inhalers demonstrated higher fine-particle fraction (FPF) values in vitro, it must be emphasized that such findings do not directly imply improved clinical efficacy or safety. According to regulatory guidelines from authorities such as the EMA and FDA, any substitution of inhalation devices requires formal pharmacokinetic and pharmacodynamic bridging studies to confirm therapeutic equivalence. Therefore, the observed differences in FPF should be interpreted with caution and must not serve as a rationale for off-label device substitution without appropriate clinical validation.

## Figures and Tables

**Table 1:** Chromatographic conditions used for the quantitative analysis of formoterol fumarate, tiotropium bromide, and glycopyrronium bromide.

Parameter	Formoterol Fumarate	Tiotropium Bromide	Glycopyrronium Bromide
**Column**	Kinetex C18 (150 mm × 4.6 mm, 5 µm)	Kinetex C18 (150 mm × 4.6 mm, 5 µm)	Kinetex C18 (150 mm × 4.6 mm, 5 µm)
**Column temperature**	30 °C	20 °C	20 °C
**Mobile phase composition**	A: 10 mM NH_4_FA + 0.1% FA (57.5%)	A: 10 mM NH_4_FA + 0.1% FA (60.0%)	A: H_2_O + 10 mM NH_4_FA + 0.1% FA (55.0%)
	B: MeOH + 0.1% FA (42.5%)	B: MeOH + 0.1% FA (40.0%)	B: MeOH + 0.1% FA (45.0%)
**Flow rate**	1.0 mL/min	1.0 mL/min	1.0 mL/min
**Injection volume**	30 µL	30 µL	30 µL
**Run time**	7.5 min	3.0 min	5.0 min
**Detection wavelength**	230 nm	230 nm	230 nm

**Abbreviations:** MeOH—methanol; FA—formic acid; NH_4_FA—ammonium formate; H_2_O—water.

**Table 2:** Delivered dose (µg) and standard deviation (SD) for formoterol, glycopyrronium, and tiotropium across three DPI devices (Aerolizer, Breezhaler, Handihaler) at different flow rates.

Inhaler	Flow Rate [L/min]	Formoterol		Glycopyrronium		Tiotropium	
		Mean Delivered Dose [µg]	SD	Mean Delivered Dose [µg]	SD	Mean Delivered Dose [µg]	SD
**Aerolizer**	4 kPa *	9.59	0.25	25.09	1.01	10.28	0.22
	100	9.07	0.16	27.50	0.55	10.01	0.24
	60	8.46	0.17	21.82	1.38	6.46	0.34
	30	8.04	0.25	17.70	0.39	3.51	0.33
	15	7.84	0.27	12.56	0.55	1.35	0.26
**Breezhaler**	4 kPa *	10.01	0.21	25.65	0.03	11.03	0.39
	100	9.95	0.25	25.21	0.13	9.16	0.30
	60	8.72	0.11	20.54	0.39	8.46	3.67
	30	8.57	0.24	16.71	0.21	3.48	0.12
	15	7.88	0.11	13.83	0.32	1.99	0.81
**Handihaler**	4 kPa *	6.60	0.19	17.03	1.02	5.90	0.33
	100	8.60	0.12	23.87	1.08	8.01	0.07
	60	7.20	0.07	16.65	0.81	6.61	0.10
	30	4.47	0.08	13.64	0.54	4.94	0.38
	15	4.21	0.10	7.41	0.73	3.09	0.32

* ≈110 L/min for Aerolizer, 115 L/min for Breezhaler, and 55 L/min for Handihaler.

**Table 3:** Fine particle dose (<5 µm) of formoterol emitted from Foradil^®^ capsules (mean ± SD, µg).

Flow [L/min]	Aerolizer		Breezhaler		Handihaler	
	Mean FPD [µg]	SD	Mean FPD [µg]	SD	Mean FPD [µg]	SD
**15**	1.16	±0.02	1.43	±0.30	0.87	±0.08
**30**	1.77	±0.02	2.09	±0.29	0.99	±0.11
**60**	3.66	±0.02	3.73	±0.28	2.98	±0.09
**100**	5.71	±0.01	5.97	±0.40	5.59	±0.09
**4 kPa** *	6.15	±0.04	6.49	±0.01	2.08	±0.01

* ≈110 L min^−1^ for Aerolizer, 115 L min^−1^ for Breezhaler, and 55 L min^−1^ for Handihaler.

**Table 4:** Fine particle dose (<5 µm) of glycopyrronium emitted from Seebri^®^ capsules (mean ± SD, µg).

Flow [L/min]	Aerolizer		Breezhaler		Handihaler	
	Mean FPD [µg]	SD	Mean FPD [µg]	SD	Mean FPD [µg]	SD
**15**	0.28	±0.05	0.44	±0.05	1.27	±0.02
**30**	1.75	±0.06	2.09	±0.04	2.62	±0.04
**60**	4.58	±0.09	5.13	±0.09	3.95	±0.06
**100**	7.32	±0.10	6.39	±0.05	4.76	±0.09
**4 kPa** *	7.85	±0.16	8.24	±0.11	3.30	±0.07

* ≈110 L min^−1^ for Aerolizer, 115 L min^−1^ for Breezhaler, and 55 L min^−1^ for Handihaler.

**Table 5:** Fine particle dose (<5 µm) of tiotropium emitted from Spiriva^®^ capsules (mean ± SD, µg).

Flow [L/min]	Aerolizer		Breezhaler		Handihaler	
	Mean FPD [µg]	SD	Mean FPD [µg]	SD	Mean FPD [µg]	SD
**15**	6.57	± 0.14	7.09	± 0.10	3.34	± 0.08
**30**	12.33	± 0.12	11.51	± 0.18	7.69	± 0.11
**60**	16.73	± 0.23	15.43	± 0.21	10.88	± 0.20
**100**	22.21	± 0.34	19.90	± 0.23	19.47	± 0.24
**4 kPa** *	22.58	± 0.23	22.05	± 0.33	10.88	± 0.13

* ≈110 L min^−1^ for Aerolizer, 115 L min^−1^ for Breezhaler, and 55 L min^−1^ for Handihaler.

## Data Availability

The original contributions presented in this study are included in the article/supplementary material. Further inquiries can be directed to the corresponding author.

## Supplementary Materials

The following supporting information can be downloaded at: https://www.mdpi.com/article/10.3390/pharmaceutics17091089/s1, Figure S1: Statistical analysis of formoterol at 15 L/min (ANOVA and Tukey HSD); Table S1: ANOVA and Tukey HSD results for formoterol at 15 L/min; Figure S2: Statistical analysis of formoterol at 30 L/min (ANOVA and Tukey HSD); Table S2: ANOVA and Tukey HSD results for formoterol at 30 L/min; Figure S3: Statistical analysis of formoterol at 60 L/min (ANOVA and Tukey HSD); Table S3: ANOVA and Tukey HSD results for formoterol at 60 L/min; Figure S4: Statistical analysis of formoterol at 100 L/min (ANOVA and Tukey HSD); Table S4: ANOVA and Tukey HSD results for formoterol at 100 L/min; Figure S5: Statistical analysis of formoterol at 4 kPa (ANOVA and Tukey HSD); Table S5: ANOVA and Tukey HSD results for formoterol at 4 kPa; Figure S6: Statistical analysis of glycopyrronium at 15 L/min (ANOVA and Tukey HSD); Table S6: ANOVA and Tukey HSD results for glycopyrronium at 15 L/min; Figure S7: Statistical analysis of glycopyrronium at 30 L/min (ANOVA and Tukey HSD); Table S7: ANOVA and Tukey HSD results for glycopyrronium at 30 L/min; Figure S8: Statistical analysis of glycopyrronium at 60 L/min (ANOVA and Tukey HSD); Table S8: ANOVA and Tukey HSD results for glycopyrronium at 60 L/min; Figure S9: Statistical analysis of glycopyrronium at 100 L/min (ANOVA and Tukey HSD); Table S9: ANOVA and Tukey HSD results for glycopyrronium at 100 L/min; Figure S10: Statistical analysis of glycopyrronium at 4 kPa (ANOVA and Tukey HSD); Table S10: ANOVA and Tukey HSD results for glycopyrronium at 4 kPa; Figure S11: Statistical analysis of tiotropium at 15 L/min (ANOVA and Tukey HSD); Table S11: ANOVA and Tukey HSD results for tiotropium at 15 L/min; Figure S12: Statistical analysis of tiotropium at 30 L/min (ANOVA and Tukey HSD); Table S12: ANOVA and Tukey HSD results for tiotropium at 30 L/min; Figure S13: Statistical analysis of tiotropium at 60 L/min (ANOVA and Tukey HSD); Table S13: ANOVA and Tukey HSD results for tiotropium at 60 L/min; Figure S14: Statistical analysis of tiotropium at 100 L/min (ANOVA and Tukey HSD); Table S14: ANOVA and Tukey HSD results for tiotropium at 100 L/min; Figure S15: Statistical analysis of tiotropium at 4 kPa (ANOVA and Tukey HSD); Table S15: ANOVA and Tukey HSD results for tiotropium at 4 kPa.
