# Supplementary material for: Comparative In Vitro Deposition Analysis of Formoterol, Glycopyrronium, and Tiotropium Delivered via Capsule-Based DPI
Source: Pharmaceutics. 2025 Aug 22;17(9):1089. doi: 10.3390/pharmaceutics17091089 (PMC12472434; doi:10.3390/pharmaceutics17091089)
Supplement: Supplementary file 1 [file pharmaceutics-17-01089-s001.zip › pharmaceutics-3800606-supplementary.pdf]

pharmaceutics

MDPI

Article: Supplementary Material

Comparative In Vitro Deposition Analysis of Formoterol, Glycopyrronium and Tiotropium Delivered via Capsule-Based DPI

Adam Sikora [1,2]*, Joanna Chałupka [1,2], Kinga Lewandowska [1], Paulina Drapińska [2] and Michał Piotr Marszałł [2]

[1] Department of Medicinal Chemistry, Faculty of Pharmacy, Collegium Medicum in Bydgoszcz, Nicolaus Copernicus University in Toruń, Dr. A. Jurasza 2, 85-089 Bydgoszcz, Poland

[2] Department of Pharmaceutical Technology, Faculty of Pharmacy, Medical Biotechnology and Laboratory Medicine, Pomeranian Medical University in Szczecin, 71-251 Szczecin, Poland

adam.sikora@cm.umk.pl (A.S.); joanna.chalupka@cm.umk.pl (J.C.); kinga.lewandowska@cm.umk.pl (K.L.); paulina.drapinska@pum.edu.pl (P.D), and mmars@cm.umk.pl (M.P.M)
* Correspondence: adam.sikora@cm.umk.pl; Tel.: (+48 52-585-3532)

1. Statistical Analysis for Formoterol

1.1. Flow rate: 15 l/min

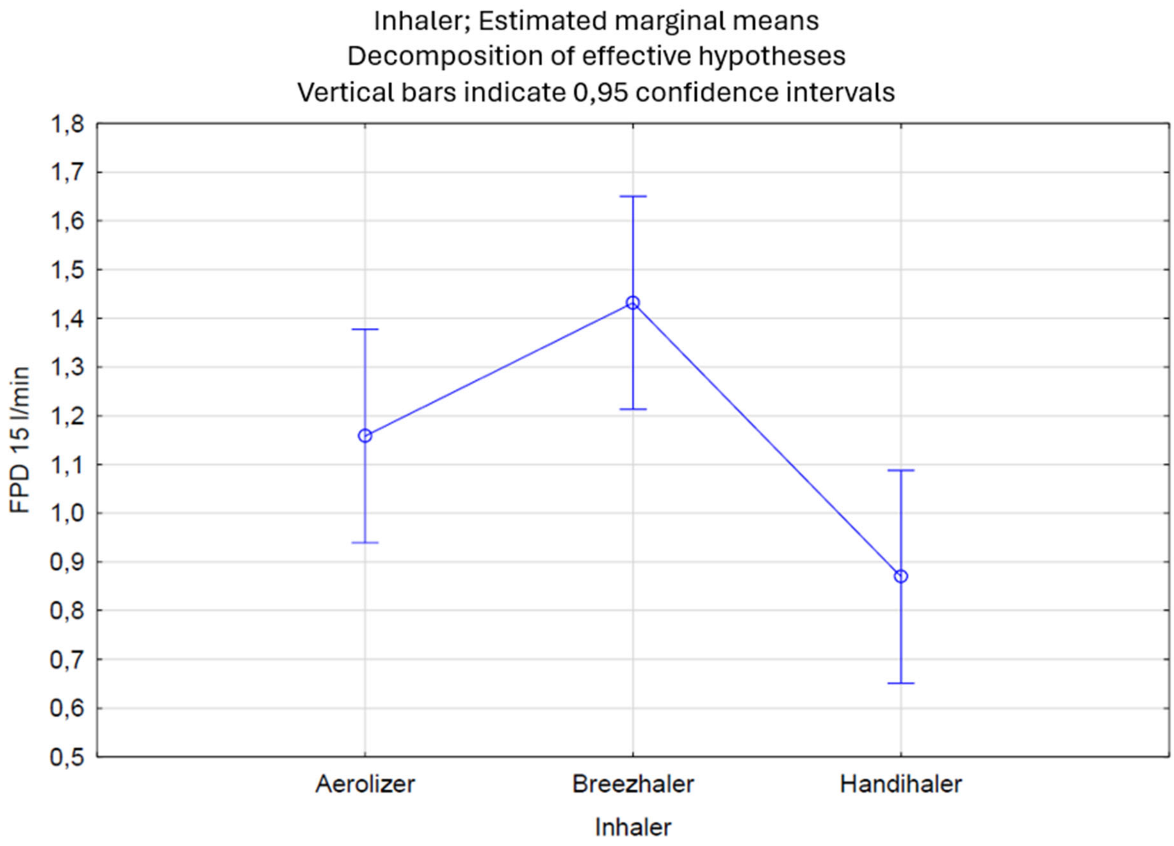

Figure S1: Statistical analysis of formoterol at 15 L/min (ANOVA and Tukey HSD)

 https://doi.org/10.3390/pharmaceutics17091089

Table S1: ANOVA and Tukey HSD results for formoterol at 15 L/min

Effect	One-dimensional significance tests, effect sizes and powers for FPD 15 l/min<br>Parametrisation with sigma-constraints<br>Decomposition of effective hypotheses				
	SS	Degrees of freedom	MS	F	p
Free expression	11.97083	1	11.97083	499.7032	0
Inhaler	0.47437	2	0.23719	9.9010	0.01257
Error	0.14374	6	0.02396		

Effect	One-dimensional significance tests, effect sizes and powers for FPD 15 l/min<br>Parametrisation with sigma constraints<br>Decomposition of effective hypotheses		
	Partial eta-squared	Non-centrality	Observed power (alpha=0.05)
Free expression	0.988135	499.7032	1.00000
Inhaler	0.767459	19.8019	0.865733
Error			

Subclass	Tukey HSD test; variable FPD 15 l/min<br>Approximate probabilities for post hoc tests<br>Error: Intergroup MS = 0.02396, df = 6.0000			
	Inhaler	1<br>1.1584	2<br>1.4319	3<br>0.86960
1	Aerolizer		0.156845	0.134370
2	Breezhaler	0.156845		0.010434
3	Handihaler	0.13437	0.010434	

1.2. Flow rate: 30 l/min

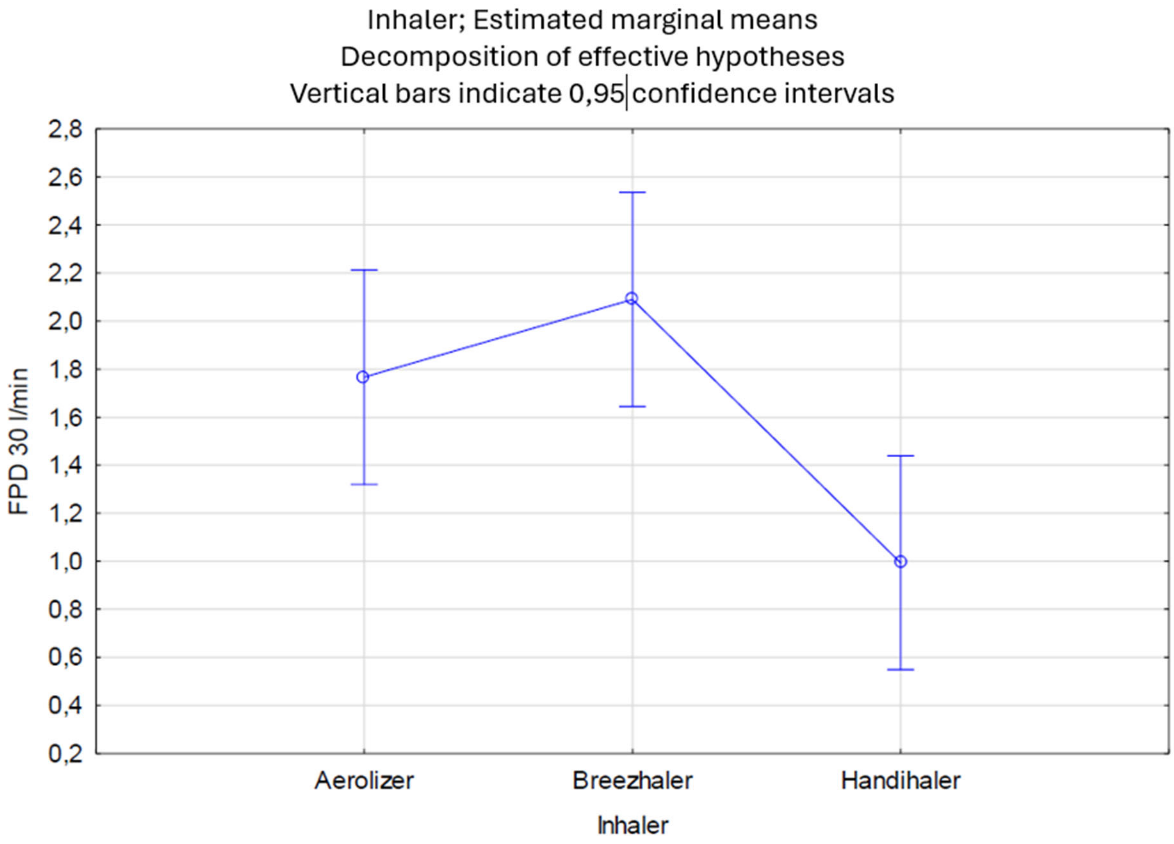

Figure S2: Statistical analysis of formoterol at 30 L/min (ANOVA and Tukey HSD)

Table S2: ANOVA and Tukey HSD results for formoterol at 30 L/min

Effect	One-dimensional significance tests, effect sizes and powers for FPD 30 l/min<br>Parametrisation with sigma constraints<br>Decomposition of effective hypotheses				
	SS	Degrees of freedom	MS	F	p
Free expression	23.52855	1	23.52855	235.9064	0
Inhaler	1.90574	2	0.95287	9.5538	0.013647
Error	0.59842	6	0.09974		

Effect	One-dimensional significance tests, effect sizes and powers for FPD 30 l/min<br>Parametrisation with sigma constraints<br>Decomposition of effective hypotheses		
	Partial eta-squared	Non-centrality	Observed power (alpha=0.05)
Free expression	0.975197	235.9064	1.00000
Inhaler	0.761029	19.1077	0.853459
Error			

Subclass	Tukey HSD test; variable FPD 30 l/min<br>Approximate probabilities for post hoc tests<br>Error: Intergroup MS = 0.09974, df = 6.0000			
	Inhaler	1<br>1.7669	2<br>2.0902	3<br>0.99345
1	Aerolizer		0.468257	0.054541
2	Breezhaler	0.468257		0.012813
3	Handihaler	0.054541	0.012813	

1.3. Flow rate: 60 l/min

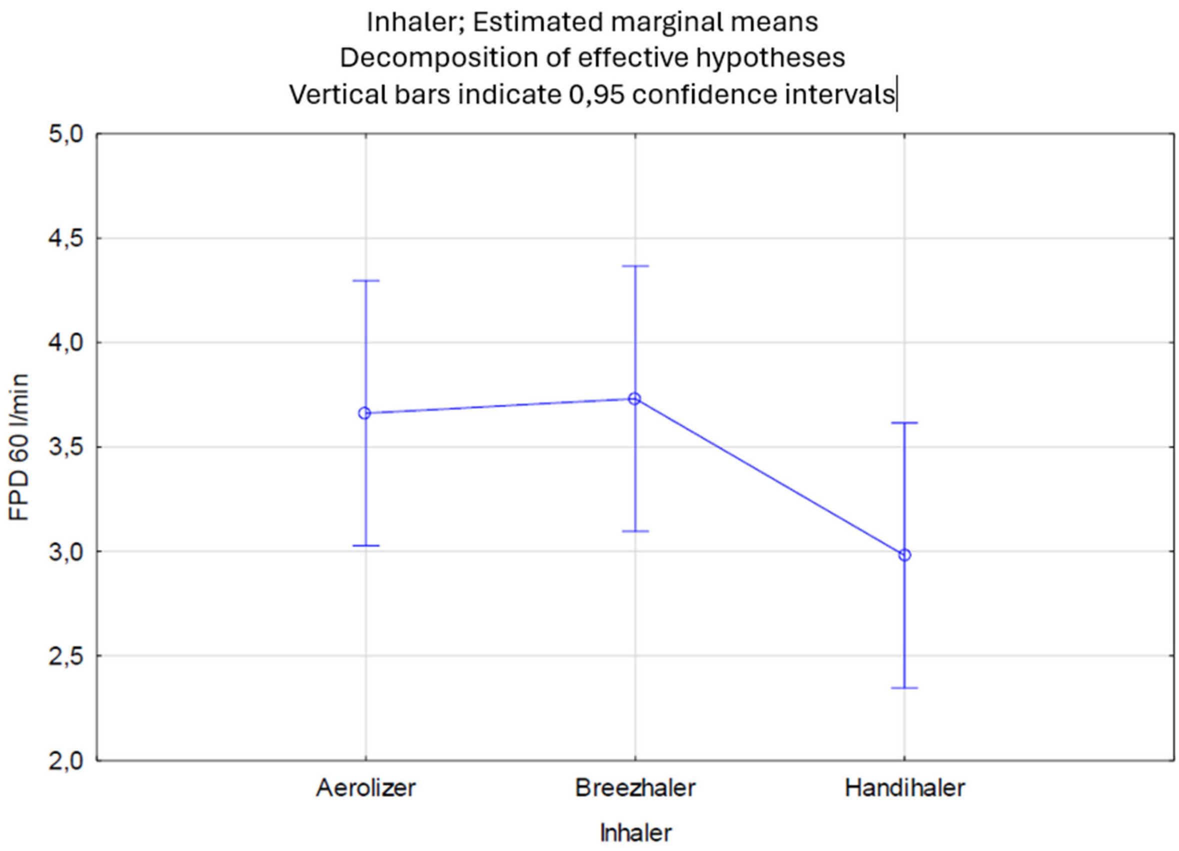

Figure S3: Statistical analysis of formoterol at 60 L/min (ANOVA and Tukey HSD)

Table S3: ANOVA and Tukey HSD results for formoterol at 60 L/min

Effect	One-dimensional significance tests, effect sizes and powers for FPD 60 l/min<br>Parametrisation with sigma constraints<br>Decomposition of effective hypotheses				
	SS	Degrees of freedom	MS	F	p
Free term	107.6365	1	107.6365	533.9864	0.0000
Inhaler	1.0301	2	0.5150	2.5551	0.157501
Error	1.2094	6	0.2016		

Effect	One-dimensional significance tests, effect sizes and powers for FPD 60 l/min<br>Parametrisation with sigma constraints<br>Decomposition of effective hypotheses		
	Partial eta-squared	Non-centrality	Observed power (alpha=0.05)
Free expression	0.9888	533.9864	1.00000
Inhaler	0.459958	5.1102	0.331420
Error			

Subclass	Tukey HSD test; variable FPD 60 l/min<br>Approximate probabilities for post hoc tests<br>Error: Intergroup MS = 0.20157, df = 6.0000			
	Inhaler	1<br>3.662	2<br>3.7313	3<br>2.9815
1	Aerolizer		0.980629	0.231164
2	Breezhaler	0.980629		0.182309
3	Handihaler	0.231164	0.182309	

1.4. Flow rate: 100 l/min

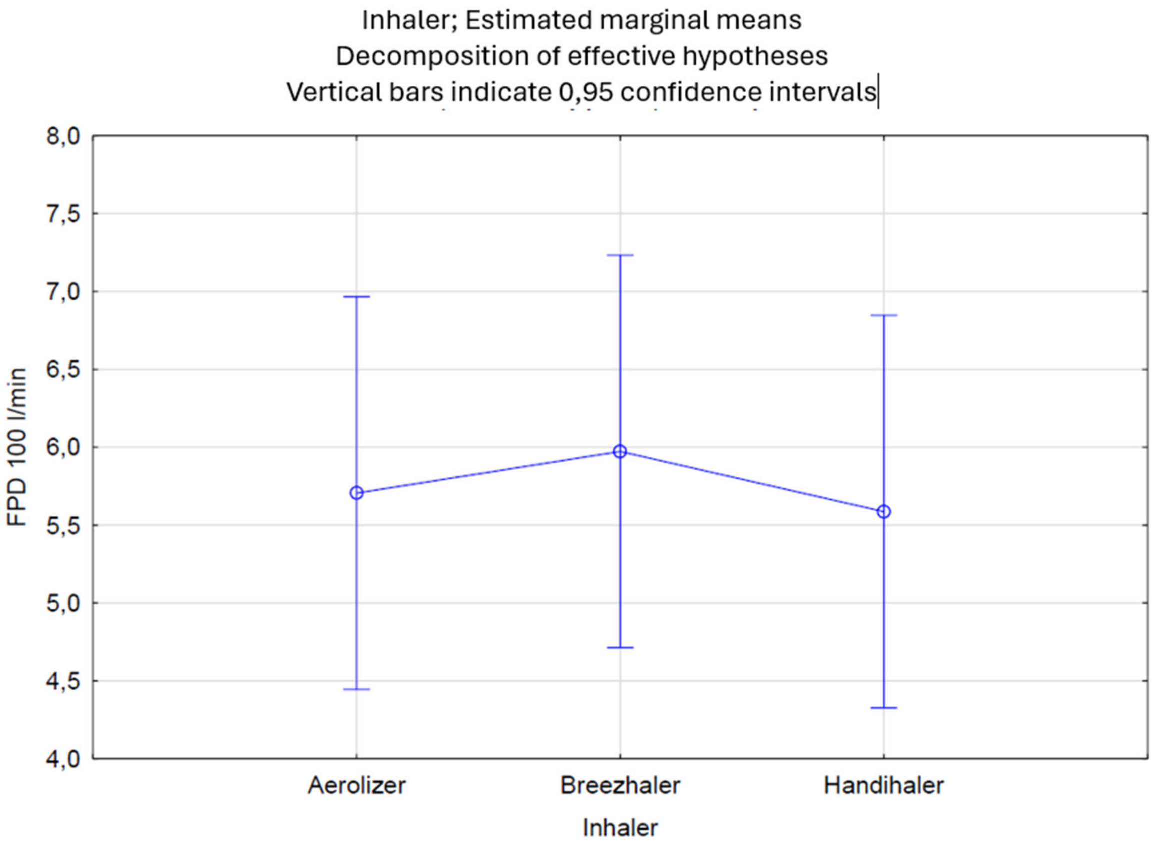

Figure S4: Statistical analysis of formoterol at 100 L/min (ANOVA and Tukey HSD)

Table S4: ANOVA and Tukey HSD results for formoterol at 100 L/min

Effect	One-dimensional significance tests, effect sizes and powers for FPD 100 l/min<br>Parametrisation with sigma constraints<br>Decomposition of effective hypotheses				
	SS	Degrees of freedom	MS	F	p
Free expression	298.1104	1	298.1104	374.5990	0
Inhaler	0.2343	2	0.1172	0.1472	0.866129
Error	4.7749	6	0.7958		

Effect	One-dimensional significance tests, effect sizes and powers for FPD 100 l/min<br>Parametrisation with sigma constraints<br>Decomposition of effective hypotheses		
	Partial eta-squared	Non-centrality	Observed power (alpha=0.05)
Free expression	0.984235	374.5990	1.00000
Inhaler	0.046778	0.2944	0.064208
Error			

Subclass	Tukey HSD test; variable FPD 100 l/min<br>Approximate probabilities for post hoc tests<br>Error: Intergroup MS = 0.79581, df = 6.0000			
	Inhaler	1<br>5.7060	2<br>5.9729	3<br>5.5870
1	Aerolizer		0.929614	0.985513
2	Breezhaler	0.929614		0.860108
3	Handihaler	0.985513	0.860108	

1.5. Flow rate: 4 kPa*

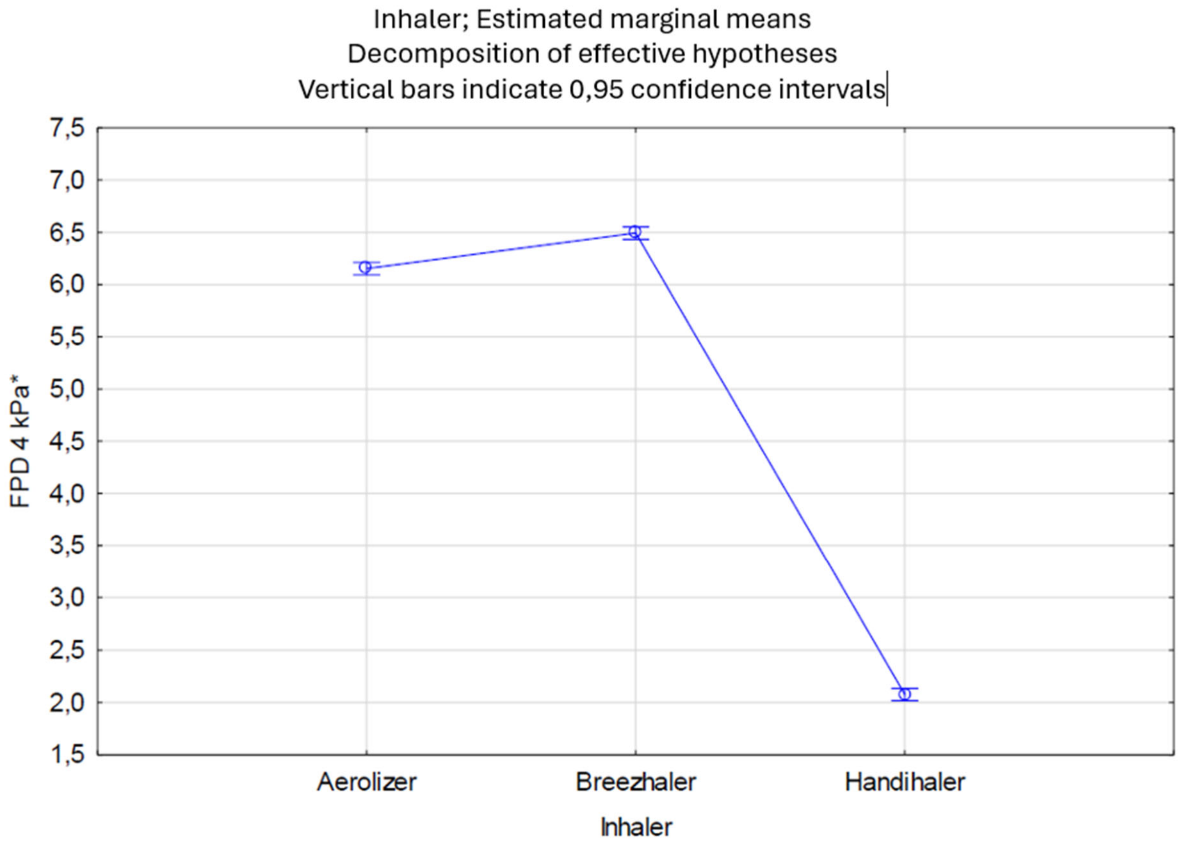

Figure S5: Statistical analysis of formoterol at 4 kPa (ANOVA and Tukey HSD)

Table S5: ANOVA and Tukey HSD results for formoterol at 4 kPa

Effect	One-dimensional significance tests, effect sizes and powers for FPD 4 kPa* Parametrisation with sigma constraints Decomposition of effective hypotheses				
	SS	Degrees of freedom	MS	F	p
Free term	216.7494	1	216.7494	122670.0	0
Inhaler	36.2354	2	18.1177	10,253.8	0.0000
Error	0.0106	6	0.0018		

Effect	One-dimensional significance tests, effect sizes and powers for FPD 4 kPa* Parametrisation with sigma constraints Decomposition of effective hypotheses		
	Partial eta-squared	Non-centrality	Observed power (alpha=0.05)
Free expression	0.999951	122670	1
Inhaler	0.999708	20,507.5	1.00000
Error			

Subclass	Tukey HSD test; variable FPD 4 kPa* Approximate probabilities for post hoc tests Error: Intergroup MS = 0.00177, df = 6.0000			
	Inhaler	1 6.1523	2 6.4934	3 2.0767
1	Aerolizer		0.000353	0.000227
2	Breezhaler	0.000353		0.000227
3	Handihaler	0.000227	0.000227	

2. Statistical Analysis for glycoppyronium

2.1. Flow rate: 15 l/min

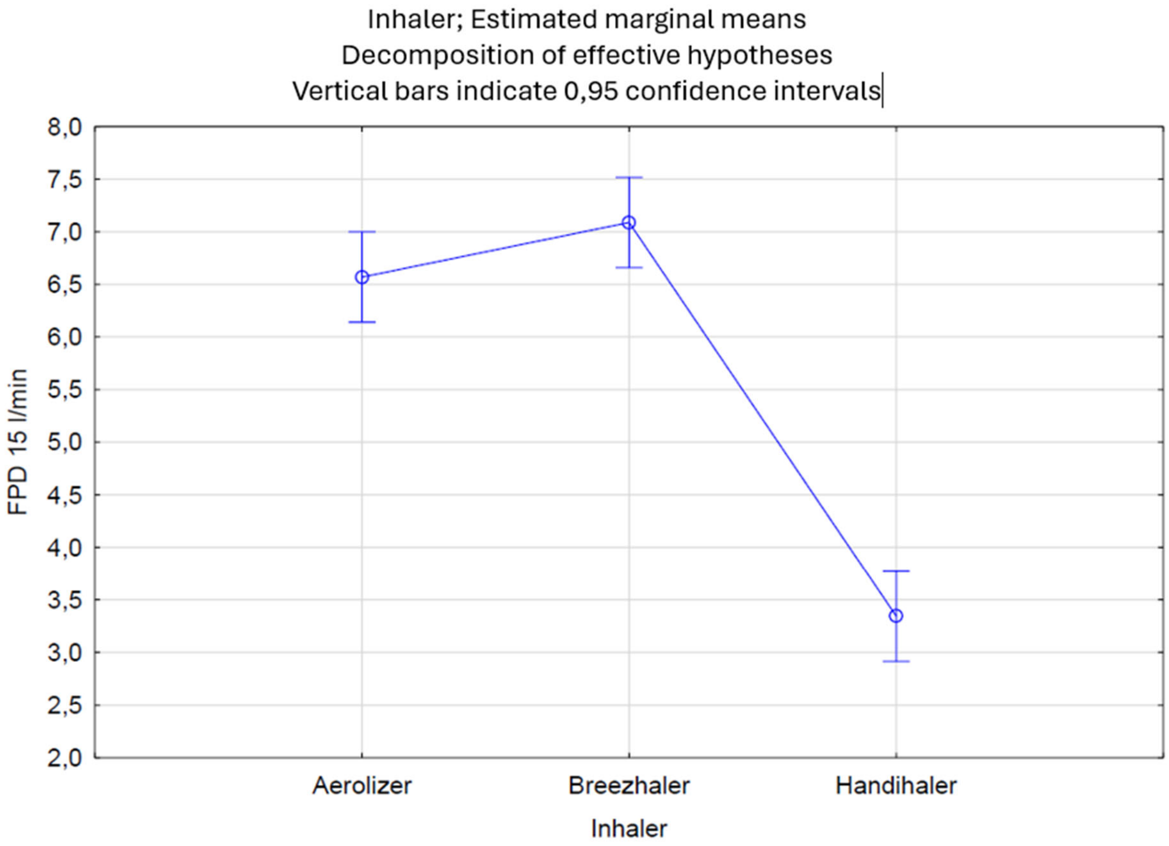

Figure S6: Statistical analysis of glycopyrronium at 15 L/min (ANOVA and Tukey HSD)

Table S6: ANOVA and Tukey HSD results for glycopyrronium at 15 L/min

Effect	One-dimensional significance tests, effect sizes and powers for FPD 15 l/min<br>Parametrisation with sigma constraints<br>Decomposition of effective hypotheses				
	SS	Degrees of freedom	MS	F	p
Free word	289.0373	1	289.0373	3143.91	0
Inhaler	24.6889	2	12.3445	134.273	0.0000
Error	0.5516	6	0.0919		

Effect	One-dimensional significance tests, effect sizes and powers for FPD 15 l/min<br>Parametrisation with sigma constraints<br>Decomposition of effective hypotheses		
	Partial eta-squared	Non-centrality	Observed power (alpha=0.05)
Free expression	0.998095	3143.91	1.00000
Inhaler	0.978146	268.546	1.000000
Error			

Subclass	Tukey HSD test; variable FPD 15 l/min<br>Approximate probabilities for post hoc tests<br>Error: Intergroup MS = 0.09194, df = 6.0000			
	Inhaler	1<br>6.5692	2<br>7.0880	3<br>3.3439
1	Aerolizer		0.171090	0.000238
2	Breezhaler	0.171090		0.000229
3	Handihaler	0.000238	0.000229	

2.2. Flow rate: 30 l/min

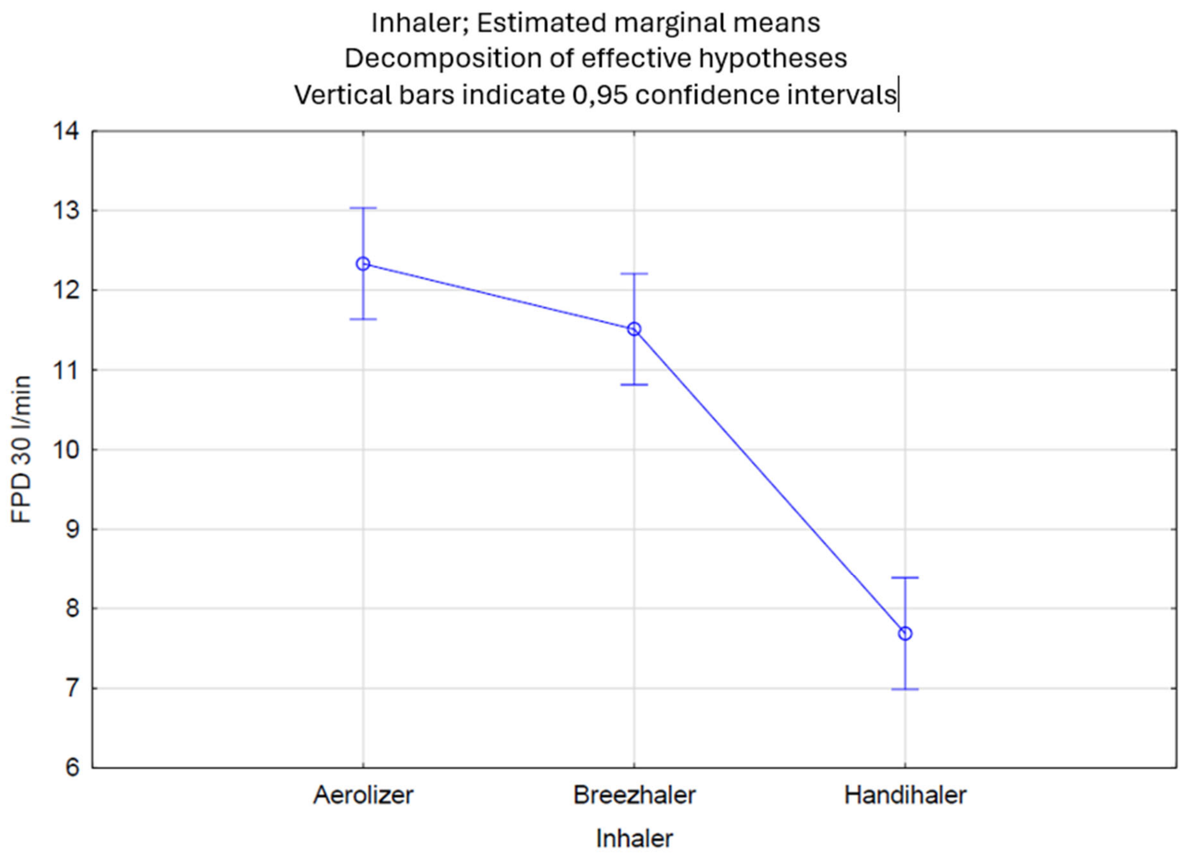

Figure S7: Statistical analysis of glycopyrronium at 30 L/min (ANOVA and Tukey HSD)

Table S7: ANOVA and Tukey HSD results for glycopyrronium at 30 L/min

Effect	One-dimensional significance tests, effect sizes and powers for FPD 30 l/min<br>Parametrisation with sigma-constraints<br>Decomposition of effective hypotheses				
	SS	Degrees of freedom	MS	F	p
Free expression	994.2130	1	994.2130	4065.086	0
Inhaler	36.8962	2	18.4481	75.43	0.000056
Error	1.4674	6	0.2446		

Effect	One-dimensional significance tests, effect sizes and powers for FPD 30 l/min<br>Parametrisation with sigma constraints<br>Decomposition of effective hypotheses		
	Partial eta-squared	Non-centrality	Observed power (alpha=0.05)
Free expression	0.998526	4065.086	1.00000
Inhaler	0.961749	150.859	1.000000
Error			

Subclass	Tukey HSD test; variable FPD 30 l/min<br>Approximate probabilities for post hoc tests<br>Error: Intergroup MS = 0.24457, df = 6.0000			
	Inhaler	1<br>1	2<br>11.511	3<br>7.6866
1	Aerolizer		0.184536	0.000260
2	Breezhaler	0.184536		0.000393
3	Handihaler	0.000260	0.000393	

2.3. Flow rate: 60 l/min

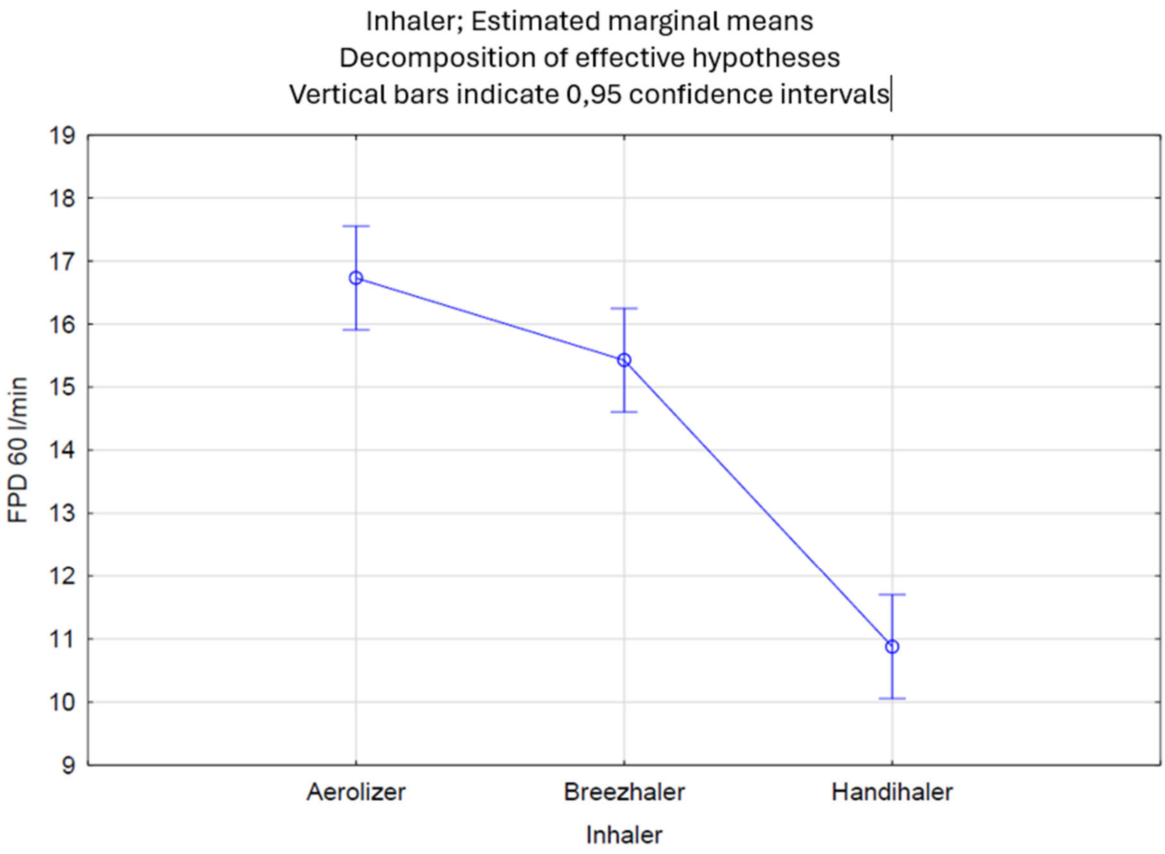

Figure S8: Statistical analysis of glycopyrronium at 60 L/min (ANOVA and Tukey HSD)

Table S8: ANOVA and Tukey HSD results for glycopyrronium at 60 L/min

Effect	One-dimensional significance tests, effect sizes and powers for FPD 60 l/min<br>Parametrisation with sigma constraints<br>Decomposition of effective hypotheses				
	SS	Degrees of freedom	MS	F	p
Free term	1852.132	1	1852.132	5488.664	0
Inhaler	56,653	2	28,327	83,944	0.000041
Error	2.025	6	0.337		

Effect	One-dimensional significance tests, effect sizes and powers for FPD 60 l/min<br>Parametrisation with sigma constraints<br>Decomposition of effective hypotheses		
	Partial eta-squared	Non-centrality	Observed power (alpha=0.05)
Free expression	0.998908	5488.664	1.00000
Inhaler	0.965495	167.888	1.000000
Error			

Subclass	Tukey HSD test; variable FPD 60 l/min<br>Approximate probabilities for post hoc tests<br>Error: Intergroup MS = 0.33745, df = 6.0000			
	Inhaler	1<br>16.731	2<br>15.427	3<br>10,878
1	Aerolizer		0.074502	0.000245
2	Breezhaler	0.074502		0.000382
3	Handihaler	0.000245	0.000382	

2.4. Flow rate: 100 l/min

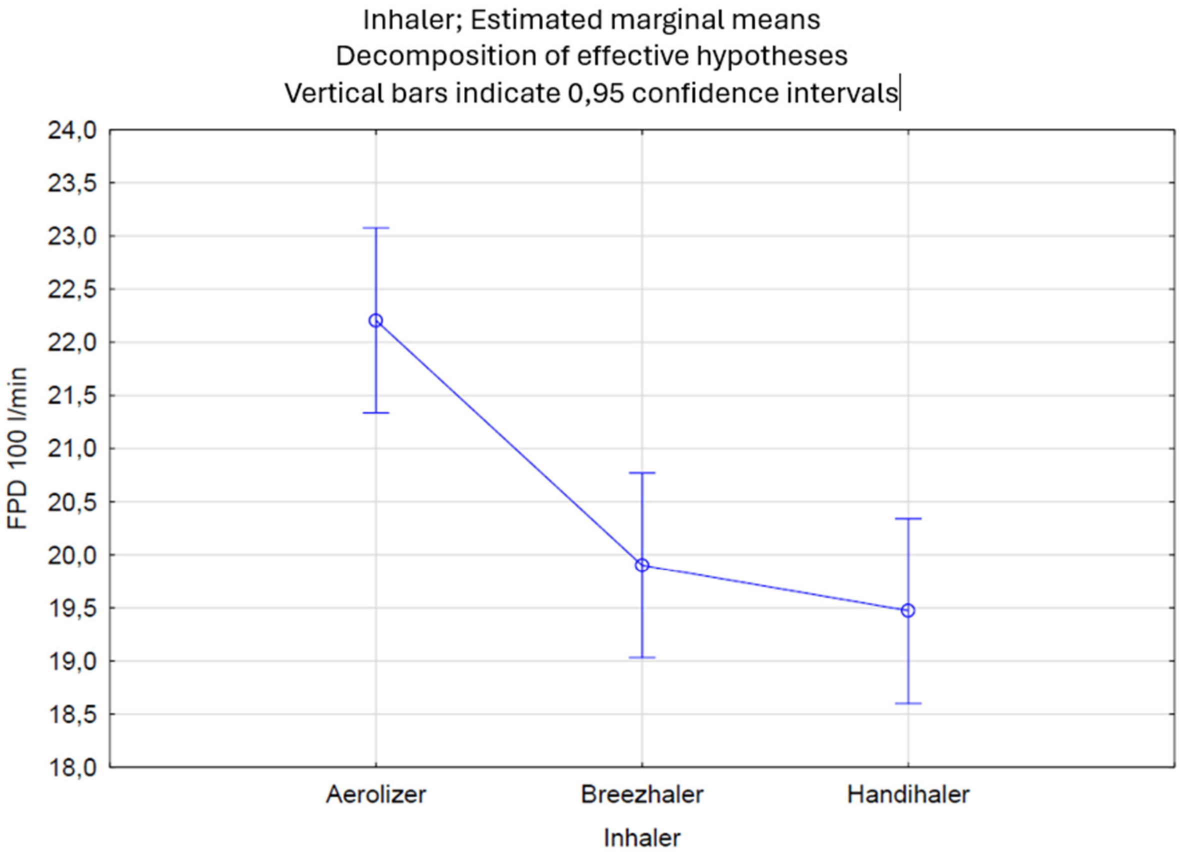

Figure S9: Statistical analysis of glycopyrronium at 100 L/min (ANOVA and Tukey HSD)

Table S9: ANOVA and Tukey HSD results for glycopyrronium at 100 L/min

Effect	One-dimensional significance tests, effect sizes and powers for FPD 100 l/min<br>Parametrisation with sigma constraints<br>Decomposition of effective hypotheses				
	SS	Degrees of freedom	MS	F	p
Free term	3791.783	1	3791.783	9994.627	0
Inhaler	12.972	2	6,486	17.097	0.003326
Error	2.276	6	0.379		

Effect	One-dimensional significance tests, effect sizes and powers for FPD 100 l/min<br>Parametrisation with sigma constraints<br>Decomposition of effective hypotheses		
	Partial eta-squared	Non-centrality	Observed power (alpha=0.05)
Free expression	0.999400	9994.627	1.00000
Inhaler	0.850723	34.194	0.981037
Error			

Subclass	Tukey HSD test; variable FPD 100 l/min<br>Approximate probabilities for post hoc tests<br>Error: Intergroup MS = 0.37938, df = 6.0000			
	Inhaler	1<br>22,206	2<br>19.90	3<br>19,472
1	Aerolizer		0.009080	0.004031
2	Breezhaler	0.00908		0.688116
3	Handihaler	0.004031	0.688116	

2.5. Flow rate: 4kPa*

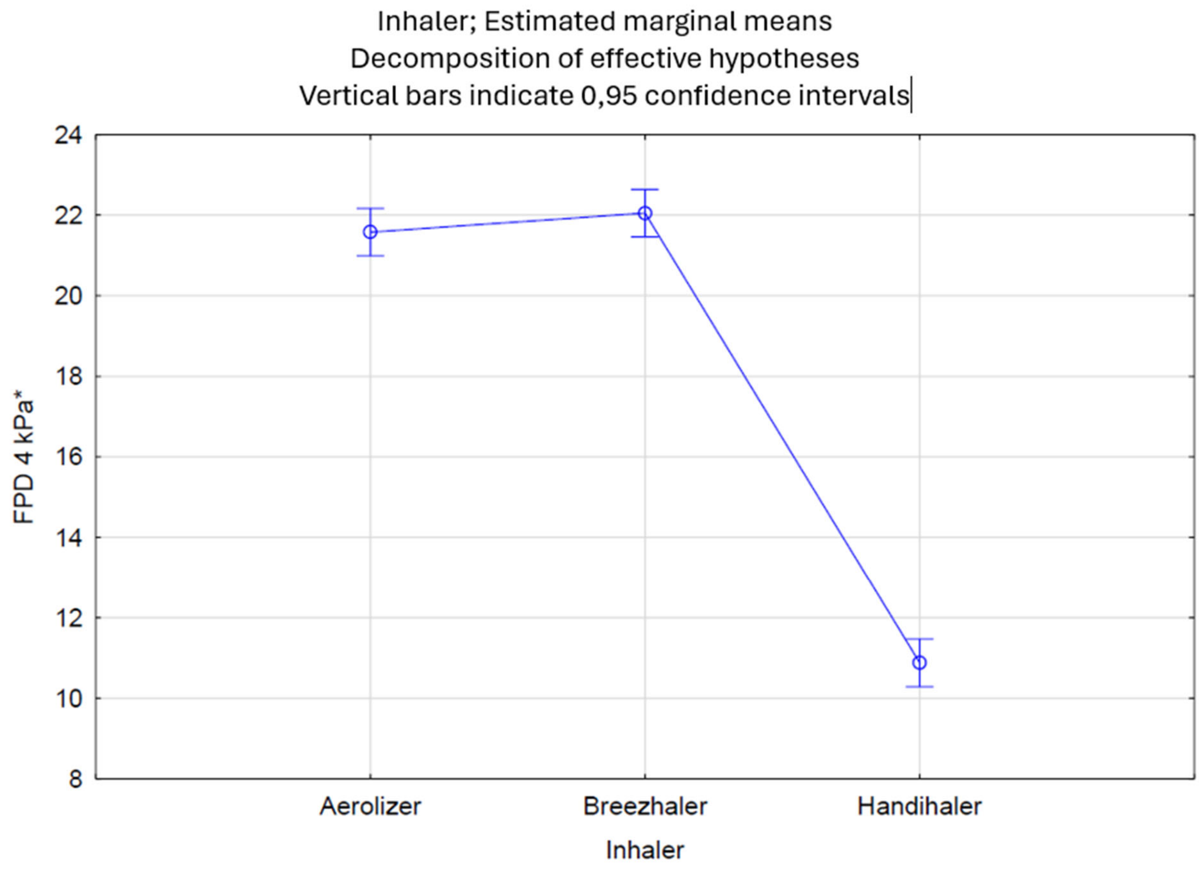

Figure S10: Statistical analysis of glycopyrronium at 4 kPa (ANOVA and Tukey HSD)

Table S10: ANOVA and Tukey HSD results for glycopyrronium at 4 kPa

Effect	One-dimensional significance tests, effect sizes and powers for FPD 4 kPa* Parametrisation with sigma constraints Decomposition of effective hypotheses				
	SS	Degrees of freedom	MS	F	p
Free term	2971.199	1	2971.199	17129.30	0
Inhaler	239.48	2	119.742	690.33	0.0000
Error	1.041	6	0.173		

Effect	One-dimensional significance tests, effect sizes and powers for FPD 4 kPa* Parametrisation with sigma constraints Decomposition of effective hypotheses		
	Partial eta-squared	Non-centrality	Observed power (alpha=0.05)
Free expression	0.999650	17129.30	1.00000
Inhaler	0.995673	1380.65	1.000000
Error			

Subclass	Tukey HSD test; variable FPD 4 kPa* Approximate probabilities for post hoc tests Error: Intergroup MS = 0.17346, df = 6.0000			
	Inhaler	1<br>21.579	2<br>22.050	3<br>10,880
1	Aerolizer		0.405049	0.000227
2	Breezhaler	0.405049		0.000227
3	Handihaler	0.000227	0.000227	

3. Statistical Analysis for Tiotropium

3.1. Flow rate: 15 l/min

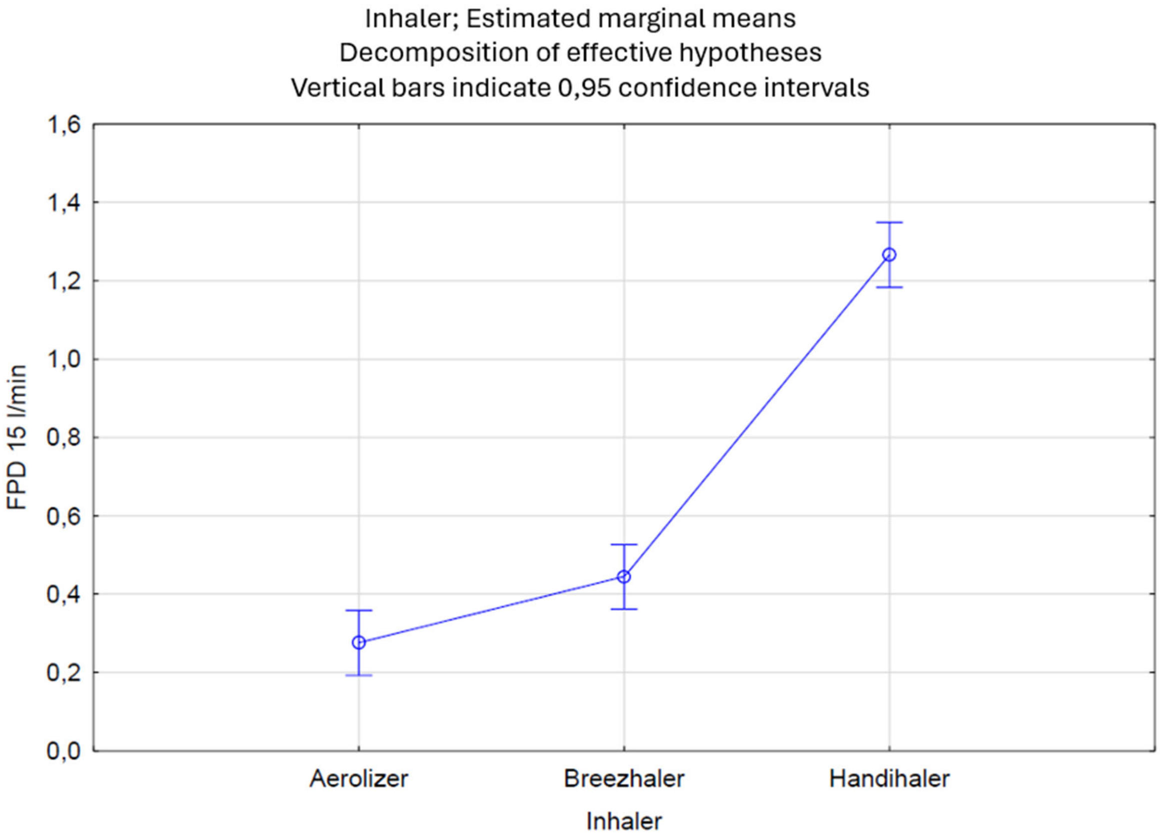

Figure S11: Statistical analysis of tiotropium at 15 L/min (ANOVA and Tukey HSD)

Table S11: ANOVA and Tukey HSD results for tiotropium at 15 L/min

Effect	One-dimensional tests of significance, effect sizes and powers for FPD 15 l/min<br>Parametrisation with sigma-constraints<br>Decomposition of effective hypotheses				
	SS	Degrees of freedom	MS	F	p
Free word	3.944963	1	3.944963	1150.065	0
Inhaler	1.686014	2	0.843007	245.76	0.000002
Error	0.020581	6	0.003430		

Effect	One-dimensional significance tests, effect sizes and powers for FPD 15 l/min<br>Parametrisation with sigma constraints<br>Decomposition of effective hypotheses		
	Partial eta-squared	Non-centrality	Observed power (alpha=0.05)
Free expression	0.994810	1150.065	1.00000
Inhaler	0.987940	491.519	1.000000
Error			

Subclass	Tukey HSD test; variable FPD 15 l/min<br>Approximate probabilities for post hoc tests<br>Error: Intergroup MS = 0.00343, df = 6.0000			
	Inhaler	1<br>0.2757	2<br>0.44410	3<br>1.2664
1	Aerolizer		0.029075	0.000227
2	Breezhaler	0.029075		0.000227
3	Handihaler	0.000227	0.000227	

3.2. Flow rate: 30 l/min

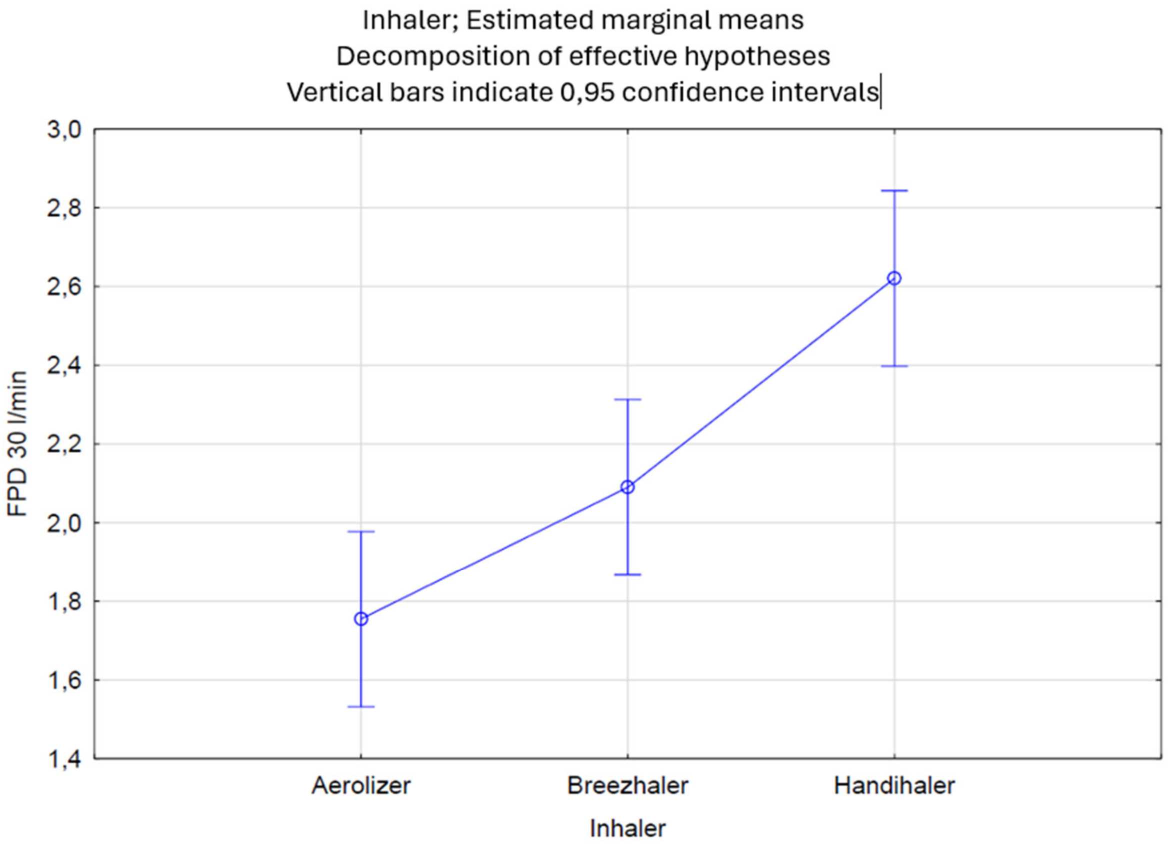

Figure S12: Statistical analysis of tiotropium at 30 L/min (ANOVA and Tukey HSD)

Table S12: ANOVA and Tukey HSD results for tiotropium at 30 L/min

Effect	One-dimensional significance tests, effect sizes and powers for FPD 30 l/min<br>Parametrisation with sigma-constraints<br>Decomposition of effective hypotheses				
	SS	Degrees of freedom	MS	F	p
Free word	41.79391	1	41.79391	1679.556	0
Inhaler	1.14352	2	0.57176	22.977	0.001540
Error	0.14930	6	0.02488		

Effect	One-dimensional significance tests, effect sizes and powers for FPD 30 l/min<br>Parametrisation with sigma constraints<br>Decomposition of effective hypotheses		
	Partial eta-squared	Non-centrality	Observed power (alpha=0.05)
Free expression	0.996440	1679.556	1.00000
Inhaler	0.884514	45.954	0.996661
Error			

Subclass	Tukey HSD test; variable FPD 30 l/min<br>Approximate probabilities for post hoc tests<br>Error: Intergroup MS = 0.02488, df = 6.0000			
	Inhaler	1<br>1.7547	2<br>2.0897	3<br>2.6205
1	Aerolizer		0.089924	0.001470
2	Breezhaler	0.089924		0.014770
3	Handihaler	0.001470	0.014770	

3.3. Flow rate: 60 l/min

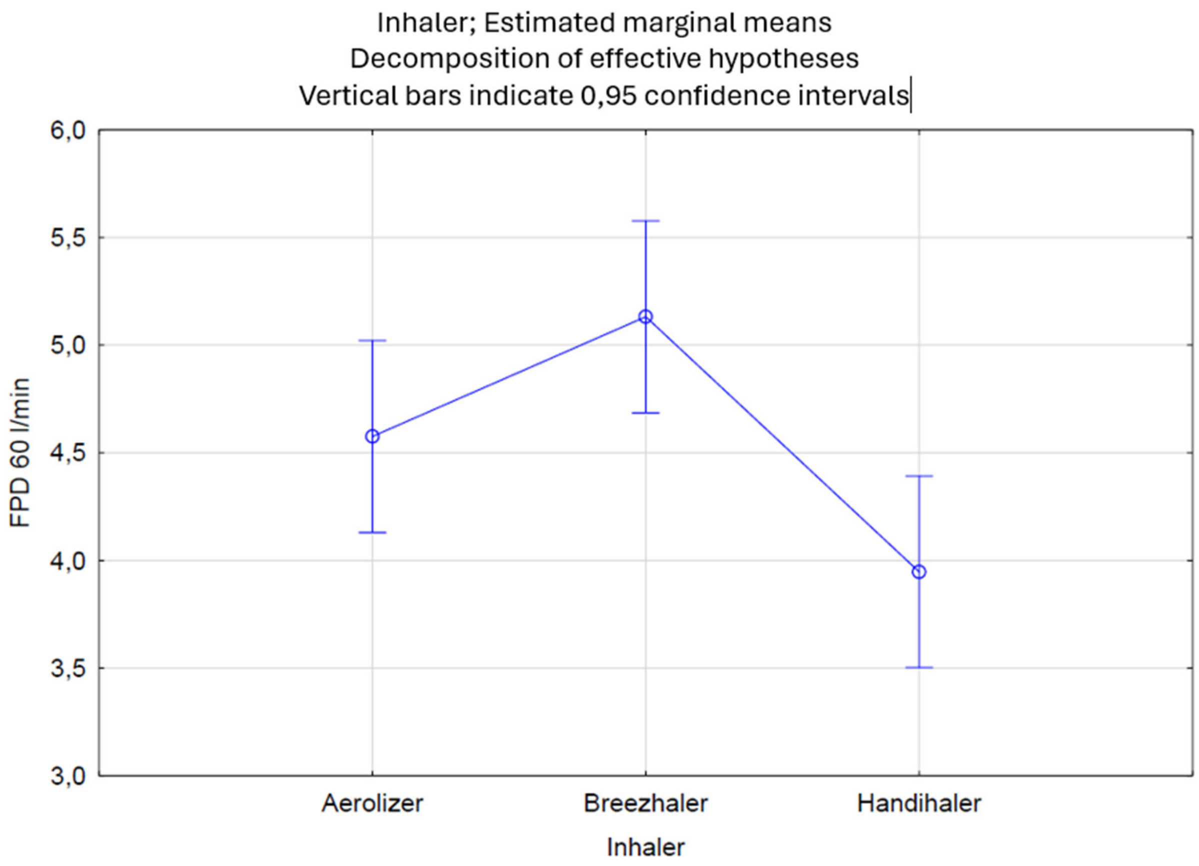

Figure S13: Statistical analysis of tiotropium at 60 L/min (ANOVA and Tukey HSD)

Table S13: ANOVA and Tukey HSD results for tiotropium at 60 L/min

Effect	One-dimensional significance tests, effect sizes and powers for FPD 60 l/min<br>Parametrisation with sigma constraints<br>Decomposition of effective hypotheses				
	SS	Degrees of freedom	MS	F	p
Free term	186.4837	1	186.4837	1877.555	0.0000
Inhaler	2.1044	2	1.0522	10.594	0.010748
Error	0.5959	6	0.0993		

Effect	One-dimensional significance tests, effect sizes and powers for FPD 60 l/min<br>Parametrisation with sigma constraints<br>Decomposition of effective hypotheses		
	Partial eta-squared	Non-centrality	Observed power (alpha=0.05)
Free expression	0.996815	1877.555	1.00000
Inhaler	0.779314	21.188	0.887515
Error			

Subclass	Tukey HSD test; variable FPD 60 l/min<br>Approximate probabilities for post hoc tests<br>Error: Intergroup MS = 0.09932, df = 6.0000			
	Inhaler	1<br>4.5761	2<br>5.1317	3<br>3.9480
1	Aerolizer		0.157823	0.110190
2	Breezhaler	0.157823		0.008936
3	Handihaler	0.110190	0.008936	

3.4. Flow rate: 100 l/min

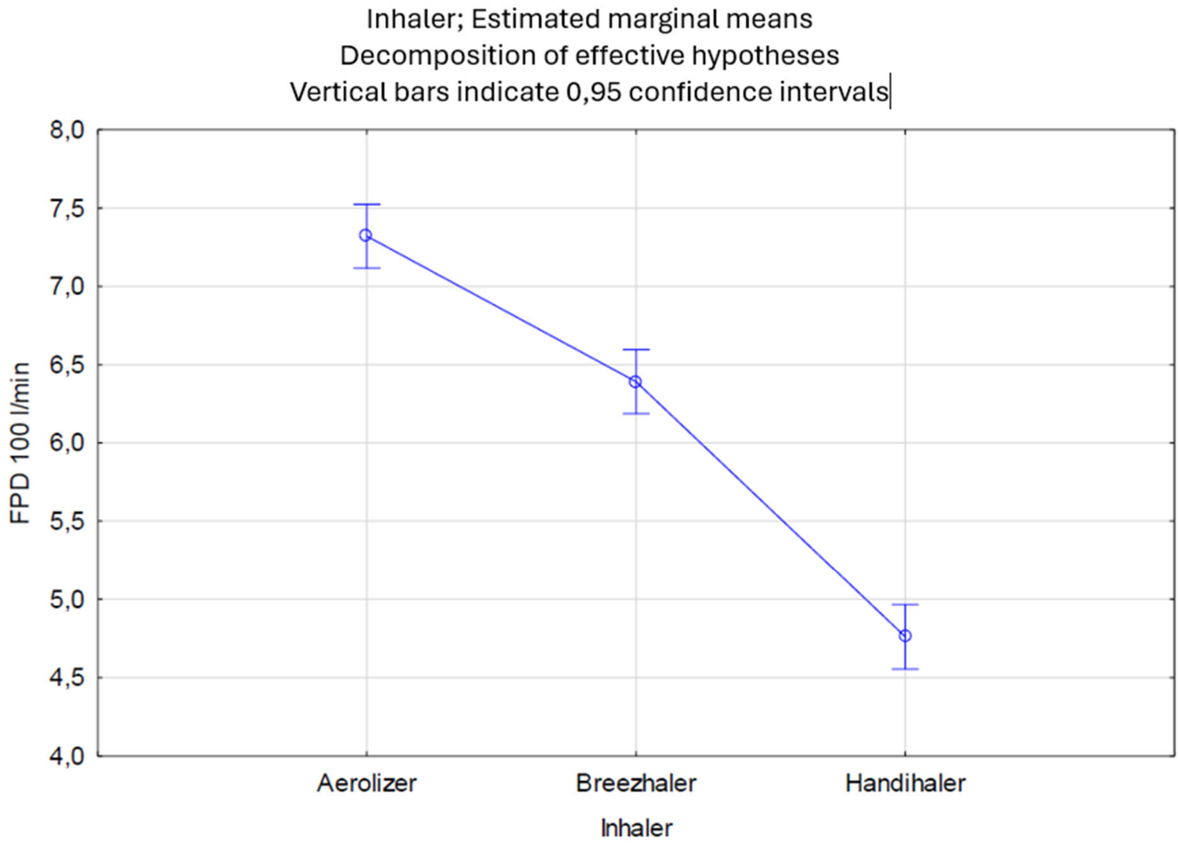

Figure S14: Statistical analysis of tiotropium at 100 L/min (ANOVA and Tukey HSD)

Table S14: ANOVA and Tukey HSD results for tiotropium at 100 L/min

Effect	One-dimensional significance tests, effect sizes and powers for FPD 100 l/min<br>Parameterisation with sigma constraints<br>Decomposition of effective hypotheses				
	SS	Degrees of freedom	MS	F	p
Free expression	341.2004	1	341.2004	16286.18	0
Inhaler	10.0802	2	5.0401	240.58	0.000002
Error	0.1257	6	0.0210		

Effect	One-dimensional significance tests, effect sizes and powers for FPD 100 l/min<br>Parametrisation with sigma constraints<br>Decomposition of effective hypotheses		
	Partial eta-squared	Non-centrality	Observed power (alpha=0.05)
Free expression	0.999632	16286.18	1.00000
Inhaler	0.987683	481.15	1.000000
Error			

Subclass	Tukey HSD test; variable FPD 100 l/min<br>Approximate probabilities for post hoc tests<br>Error: Intergroup MS = 0.02095, df = 6.0000			
	Inhaler	1<br>7.3203	2<br>6.3913	3<br>4.7599
1	Aerolizer		0.000728	0.000227
2	Breezhaler	0.000728		0.000233
3	Handihaler	0.000227	0.000233	

3.5. Flow rate: 4kPa*

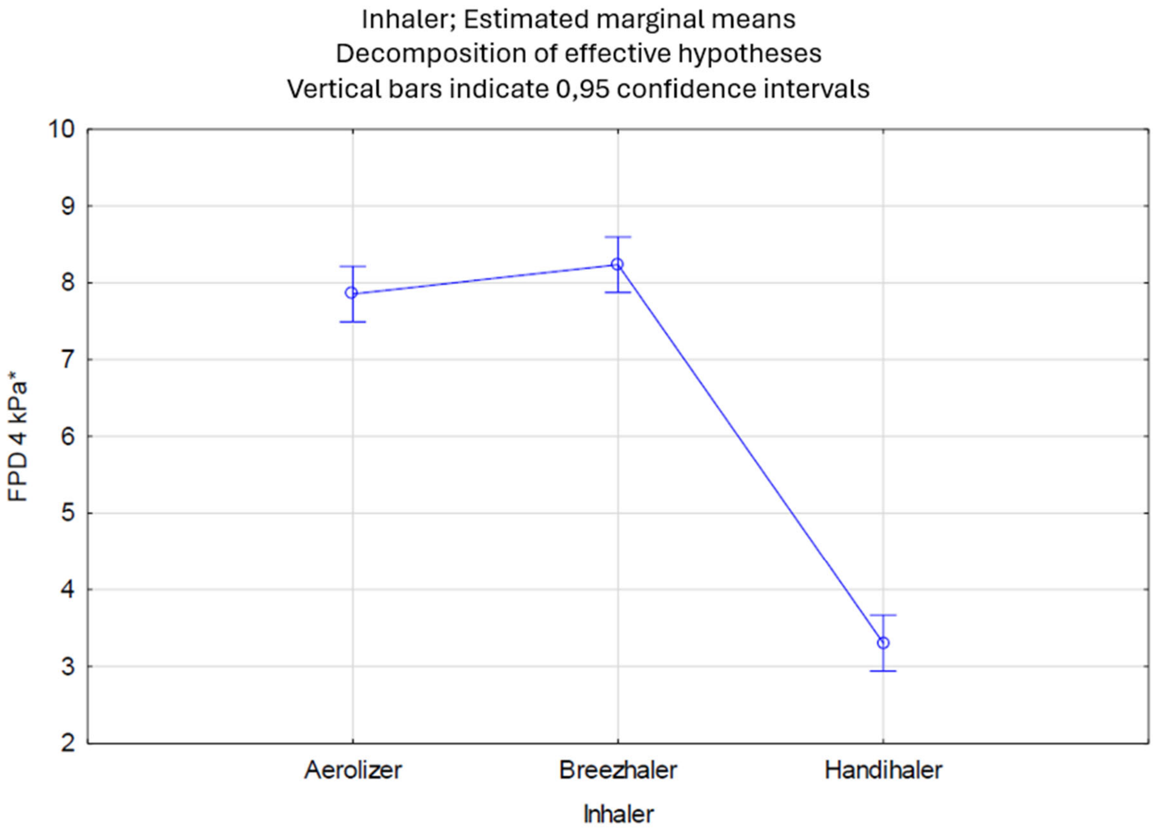

Figure S15: Statistical analysis of tiotropium at 4 kPa (ANOVA and Tukey HSD)

Table S15: ANOVA and Tukey HSD results for tiotropium at 4 kPa

Effect	One-dimensional significance tests, effect sizes and powers for FPD 4 kPa* Parametrisation with sigma constraints Decomposition of effective hypotheses				
	SS	Degrees of freedom	MS	F	p
Free term	376.1251	1	376.1251	5732.223	0
Inhaler	45.1685	2	22.5843	344.189	0.000001
Error	0.3937	6	0.0656		

Effect	One-dimensional significance tests, effect sizes and powers for FPD 4 kPa* Parametrisation with sigma constraints Decomposition of effective hypotheses		
	Partial eta-squared	Non-centrality	Observed power (alpha=0.05)
Free expression	0.998954	5732.223	1.00000
Inhaler	0.991359	688.377	1.000000
Error			

Subclass No.	Tukey HSD test; variable FPD 4 kPa* Approximate probabilities for post hoc tests Error: Intergroup MS = 0.06562, df = 6.0000			
	Inhaler	1<br>7.8539	2<br>8.2359	3<br>3.3041
1	Aerolizer		0.240004	0.000227
2	Breezhaler	0.240004		0.000227
3	Handihaler	0.000227	0.000227	
